# New 1,3-Disubstituted Benzo[*h*]Isoquinoline Cyclen-Based Ligand Platform: Synthesis, Eu^3+^ Multiphoton Sensitization and Imaging Applications

**DOI:** 10.3390/molecules26010058

**Published:** 2020-12-24

**Authors:** Sebastiano Di Pietro, Dalila Iacopini, Barbara Storti, Riccardo Nifosì, Valeria Di Bussolo, Mauro Pineschi, Aldo Moscardini, Giovanni Signore, Ranieri Bizzarri

**Affiliations:** 1 Department of Pharmacy, University of Pisa, Via Bonanno Pisano 6, 56126 Pisa, Italy; sebastiano.dipietro@unipi.it (S.D.P.); mauro.pineschi@unipi.it (M.P.); 2 Chemistry and Industrial Chemistry Department, University of Pisa, Via G. Moruzzi 3, 56126 Pisa, Italy; dalila.iacopini@phd.unipi.it (D.I.); valeria.dibussolo@unipi.it (V.D.B.); 3 CNR-NANO NEST, Scuola Normale Superiore and Istituto Nanoscienze-CNR, Piazza San Silvestro 12, 56127 Pisa, Italy; barbara.storti@nano.cnr.it (B.S.); riccardo.nifosi@nano.cnr.it (R.N.); aldo.moscardini@sns.it (A.M.); 4 Fondazione Pisana per la Scienza, Via F. Giovannini 13, 56017 San Giuliano Terme (PI), Italy; 5 Department of Surgical, Medical and Molecular Pathology, and Critical Care medicine, University of Pisa, Via Roma 55, 56126 Pisa, Italy

**Keywords:** europium complex, benzoisoquinoline, cyclen, bioprobe, structured illumination microscopy

## Abstract

The development of lanthanide-based luminescent probes with a long emission lifetime has the potential to revolutionize imaging-based diagnostic techniques. By a rational design strategy taking advantage of computational predictions, a novel, water-soluble Eu^3+^ complex from a cyclen-based ligand bearing 1,3-disubstituted benzo[h]isoquinoline arms was realized. The ligand has been obtained overcoming the lack of reactivity of position 3 of the isoquinoline moiety. Notably, steric hindrance of the heteroaromatic chromophore allowed selective and stoichiometry-controlled insertion of two or three antennas on the cyclen platform without any protection strategy. The complex bears a fourth heptanoic arm for easy conjugation to biomolecules. This new chromophore allowed the sensitization of the metal center either with one or two photons excitation. The suitability as a luminescent bioprobe was validated by imaging BMI1 oncomarker in lung carcinoma cells following an established immunofluorescence approach. The use of a conventional epifluorescence microscope equipped with a linear structured illumination module disclosed a simple and inexpensive way to image confocally Ln-bioprobes by single photon excitation in the 350–400 nm window, where ordinary confocal systems have no excitation sources.

## 1. Introduction

In the last few years, luminescent lanthanide (Ln) complexes (mainly based on Eu^3+^ and Tb^3+^) have attracted considerable attention as probes for a wide range of bioapplications [1,2]. Indeed luminescent Ln-complexes have many unique properties compared to traditional fluorescent bioprobes [3]. The large Stokes’ shift and sharp emission profiles, for example, enable easy spectral decoupling of excitation from emission and favour the applications of lanthanide bio-probes in imaging techniques hampered by spectral cross-talk and bleed-through, such as Forster resonance energy transfer (FRET). The long luminescent lifetime in the μs–ms range [4], however, is perhaps the most intriguing feature of lanthanide bioprobes, since it allows for time-resolved luminescence detection to filter off fast-decaying (ps to ns) signals such as autofluorescence or residual acceptor cross-talk in time-resolved FRET (TR-FRET) spectroscopic analysis [5,6,7]. Lifetime gating of Ln-complexes has also been exploited in wide-field and confocal microscopy [6,8,9,10,11]. Owing to the emission in the red visible region (580–720 nm), Eu^3+^ complexes attract particular interests in all those multicolor imaging applications involving bright genetically-encodable probes, such as the popular toolbox of green-emitting fluorescent proteins [12].

The long lifetime of Ln luminescence stems from the partially forbidden nature of the associated 4f−4f transitions, which have nonetheless very low oscillator strengths and cannot be directly photoexcited [13] The ligand in the complex has the delicate function to collect the photoexcitation energy and, by energy transfer, to sensitize Ln to emission (antenna effect). Accordingly, much interest has been devoted to the sensitization mechanism of aromatic ligands characterized by intense π-π* excitation transition [14]. The most common energy migration path goes through the long-lived triplet states (T_n_) of the ligands. Thus, the first requirement to design an effective photoluminescent Ln-complex is the presence of suitable triplet energy level(s) in the ligand(s) to funnel energy onto the metal ion [15]. A general consensus states that the lowest T_n_ energy should be 2500–3500 cm^−1^ above the main emitting level of Ln, to ensure unidirectional energy transfer from ligand to the metal ion and to maximize the sensitization efficiency (η_sens_) [3,13]. At the same time, π-systems display efficient intersystem crossing (ISC) when the energy difference between the singlet and the triplet state is above 5000 cm^−1^ [16]. Since the fundamental emitting transition of Eu^3+^ (^5^D_0_–^7^F_0_) is about 12,300 cm^−1^, this implies that the long-wavelength absorption edge of the π-π* transition must not overcome 385–405 nm. This poses a structural limit on the π-system of the ligand, whose conjugation cannot be arbitrarily extended to red-shift Ln-complex photoexcitation. Ways to circumvent this limit, which involve the use of two-photon excitation [4] or sensitization through the singlet excited state of the ligand [17], are still limited and often rely upon highly extended π-system edifices with poor water solubility. However, the possibility to realize dual excitation modes lanthanide probes, accurately balancing the π-conjugation/hydrophobicity properties of the ligand antenna, largely increase the versatility of the system. The peculiarity of the bioenvironment poses additional strict requirements over the engineering of an effective Ln-bioprobe [18]. Among others, we should remind the following indispensable features: (1) multidentate O, N sites of coordination to afford strong complex stability at physiological pH in water media; (2) embedding of the Ln ion into a rigid cavity to minimize the nonradiative deactivation of the metal; (3) hydrophilic groups to obtain good water solubility; (4) excitation wavelength above 350 nm to avoid strong phototoxicity and UV optics [19].

**Scheme 1:**
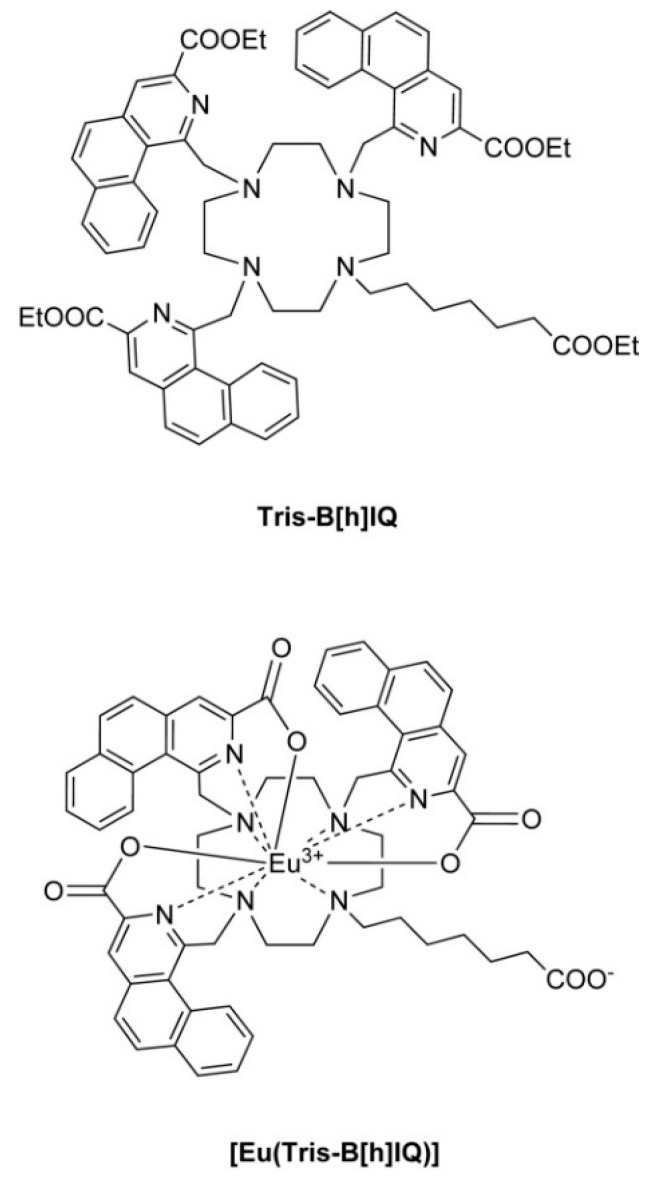
Structure of the target structures synthesized in this study: ligand **Tris-B[h]IQ** (above) and complex **[Eu(Tris-B[h]IQ]** (below).

In this work, we report the synthesis of the first example of cyclen-based 1,3-asymmetrically substituted benzo[*h*]isoquinoline ligand and the relative Eu^3+^-complex: these new sensitizing units assure good stability at physiological pH, one/two photon/s luminescence, long lifetime, and a suitable tether for bioconjugation. For the ligand, our starting point was a set of structures that substituted pyridine for isoquinoline in the well-established dipicolinic acid Ln^3+^-binding site (ligand L_1_ in ref [20]), in order to obtain the required excitation profile. We greatly improved this approach by replacing the dipicolinated scaffold by a cyclen ring (tetraazacyclododecane) bound to three benzo[*h*]isoquinolines and to one carboxyl alkyl tether enabling conjugation of biomolecules (ligand **Tris-B[h]IQ**, Scheme 1): interestingly, we achieved such a complex synthetic task, exploiting the stereolectronic properties of the ligand units, which allowed us to selectively insert three (or two) benzo[*h*]isoquinoline arms on the cyclen scaffold without any protection strategy, considerably increasing the versatility of the synthetic pathway, in the light of a possible differently substituted ligand screening.

We expected the cyclen system to increase strongly lanthanide-chelating capability and water stability compared to the dipicolinate group [21], while at the same time increasing the number of aromatic units to increase the molar extinction coefficient of the complex. In keeping with our rational design approach to novel probes for bioimaging [22,23,24,25,26,27], we first checked in silico the benzoisoquinoline aromatic system as a way to extend π-conjugation of the ligand without compromising its sensitizing capability towards Eu^3+^. This prediction was later verified experimentally. Finally, we demonstrated that our complex can be effectively utilized in immunofluorescence assays in tandem with structured illumination microscopy (SIM) [28,29]. Our SIM system combines excellent 3D sectioning capability of the sample with excitation in the 350–380 nm window, a feature rarely found in conventional laser scanning confocal microscopes. Additionally, the fast raster-scanning mode of confocal microscopes is poorly suited to Ln imaging, owing to pixel dwell times <100 μs that do not allow for the full collection of the emitted luminescence. This issue is addressed by our SIM system, whose camera detection allows for parallelized luminescence collection in all pixels with integration times tailored to the Ln luminescence decay.

## 2. Results and Discussion

### 2.1. Computational Calculations

**Figure 1:**
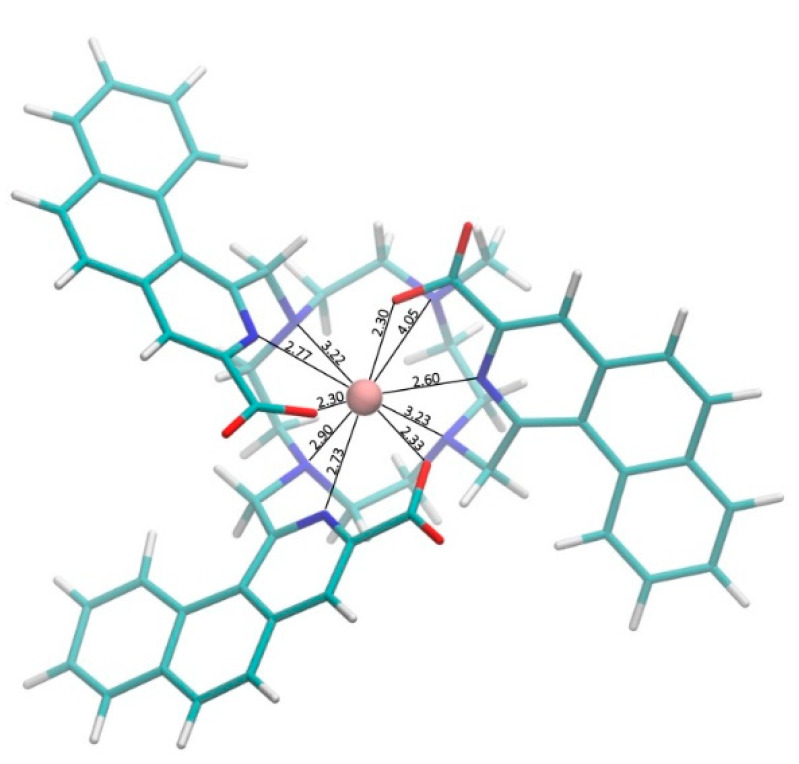
3D geometry from DFT optimization. The relevant distances of the ligand to Eu^3+^ are reported in Å. The picture was produced using VMD [32].

To explore the photophysical properties of our complex, we performed a computational study of the structure and absorption spectrum of the bare complex **[Eu(Tris-B[h]IQ)]** (Scheme 1), and compared the results with the reported EuL_1_ complex [20]. Figure 1 shows the structure of **[Eu(Tris-B[h]IQ)]** obtained by density functional theory (DFT) geometry optimization, employing B3LYP as exchange and correlation potential (for other details of this and other computations see the Supporting Information).

Generally, the agreement between X-ray structures of Eu complexes and DFT predictions is reasonably good, with bond distances errors below 0.1 Å [30]. In the optimized structure, the three carboxyl groups of the benzo[*h*]isoquinolines are coordinated to Eu^3+^ with bond distances of 2.30–2.33 Å, while their nitrogen atoms lie roughly on the same plane as Eu^3+^ with distances of 2.60–2.77 Å. These distances are in the range of values for Eu-coordinated N and O atoms [31]. The distances to the cyclen nitrogen atoms span a broader range, the one at the tether site being 4.05 Å, the one opposite 2.90 Å, and the remaining two measuring intermediate 3.22–3.23 Å values.

**Figure 2:**
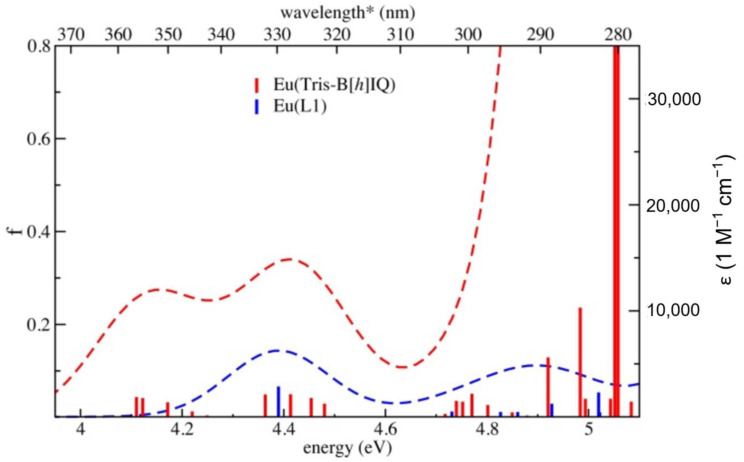
Predicted absorption spectrum of the **[Eu(Tris-B[*h*]IQ)]** complex and the benchmark complex EuL_1_ [20]. Excitation energies predicted by TD-DFT are shown as bars, with height given by the associated oscillator strength *f*. The extinction coefficient ε was calculated by summing up suitably broadened gaussian functions, one for each excitation. The top scale is the extrapolated wavelength when setting the EuL_1_ peak to the experimental 330 nm value [20].

Figure 2 reports the excitations predicted by time-dependent DFT for the two complexes. The employed CAM-B3LYP exchange and correlation potential is known to generally overestimate excitation energies [33]. However, if we focus on energy shifts between molecules in the same class, the prediction is more accurate. In this regard, we predicted a 0.27 eV red-shift for the lowest-energy band of [**Eu(Tris-B[h]IQ)]** compared to EuL_1_ [20]. Since the experimental excitation peak of EuL_1_ is at 330 nm (3.76 eV), this red-shift would correspond to experimental wavelengths of 355 nm. Thus, the extension of the aromatic π-system from isoquinoline to benzoisoquinoline was expected to effectively red-shift the ligand absorption without falling into the region where metal sensitization crumbles owing to energy back-transfer [16] This prediction was also supported by the reported experimental phosphorescence spectrum of benzoisoquinoline in water at 77 K, which shows major bands at 21,650 and 21,370 cm^−1^, i.e., in the region of highest sensitization of Eu^3+^ by triplet states [14].

### 2.2. Synthetic Strategy and Results

**Scheme 2:**
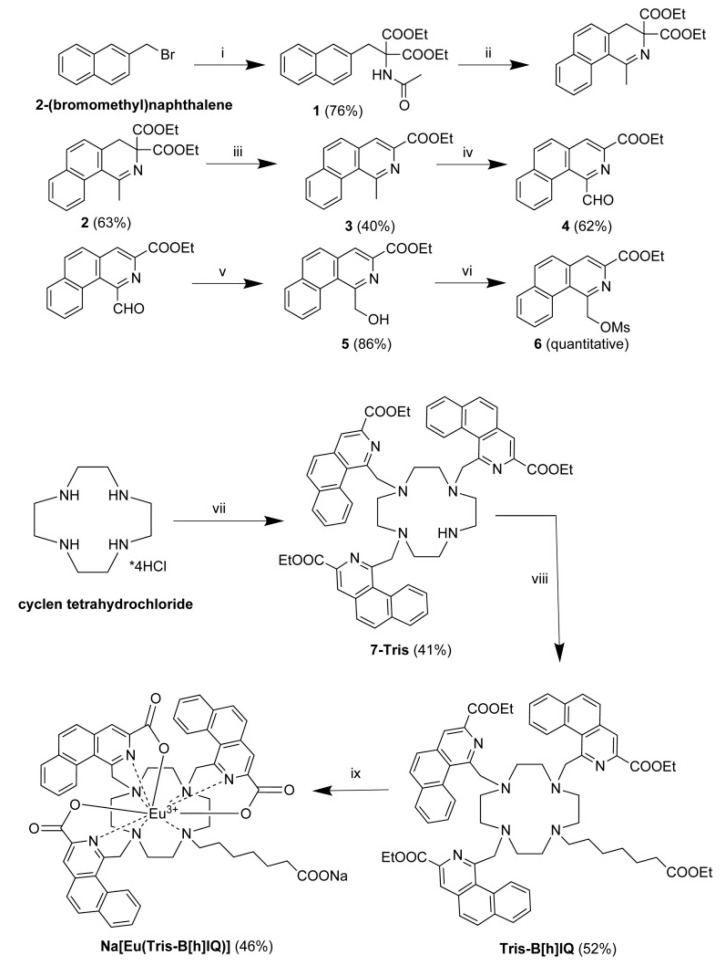
Complete synthetic pathway towards complex **[Eu(Tris-B[h]IQ]**.

Reported approaches to benzo[*h*]isoquinoline scaffold are not suitable to obtain 1,3-asymmetric compound B[h]IQ unless major modifications leading to significant increase in synthetic process complexity are implemented [34,35,36,37]. From a synthetic perspective, the major hurdle is represented by the lack of reactivity of the position 3 [38] Some examples of unsubstituted scaffolds were synthesized starting from 3-ethynyl−2-phenylpyridine through a pyrolysis process [34], or alternatively by a Pomeranz–Fritsch procedure from naphthaldehydes [35], 3,4-disubstituted (mainly with aryl fragments) benzo[*h*]isoquinolines were obtained from (1-naphthylmethyl)amine through a rhodium-catalyzed coupling reaction [37], and a 1,3-dimethyl derivative has been prepared through a Suzuki or Negishi cross-coupling followed by a cyclization reaction induced by t-BuOK [36]. In this context, we planned our synthesis by building the heteroaromatic fragment already substituted in the desired positions (Scheme 2). The synthesis started with a nucleophilic substitution between 2-(bromomethyl)naphthalene and diethyl 2-acetylaminomalonate, followed by a Bischler–Napieralski cyclization reaction in POCl_3_, which converted derivative **1** into the tricylic compound **2**.

Regioselectivity of this reaction was completely directed towards the [*h*]-isomer, in agreement with the established higher reactivity of the α position of the naphthalene ring towards aromatic electrophilic substitution reaction. The unique presence of the [*h*]-regioisomer was confirmed by HMBC and ROESY 2D-NMR studies (see Supplementary Figure S3). Next, derivative **2** was aromatized by treatment with NaI in pyridine as solvent. The 1-methyl−3-ethylester **3** was then subjected to an oxidation step, in which the methyl group on C1 is oxidized to aldehyde with selenium dioxide (intermediate **4**) and selectively reduced to primary alcohol with NaBH_4_ at −20°C to afford compound **5**. Subsequent treatment of **5** with methanesulfonyl chloride provided the aromatic antenna **6** ready to be anchored to the cyclen scaffold. Complex functionalization of aza macrocycle scaffolds usually involves tedious protection methodologies to functionalize the desired positions with high selectivity [39,40,41,42,43]. Remarkably, cyclen functionalization was obtained by direct reaction between cyclen tetrahydrochloride and **6**. Interestingly, the degree of substitution of the cyclen ring was completely controlled by the stoichiometric ratio between cyclen tetrahydrochloride and **6**: when 4 equivalents of **6** were used, the tri-substituted compound **7-Tris** was isolated as major product, while no trace of tetra-substituted compound was observed. This was likely due to the steric hindrance of **6**, which could slow down the kinetics of the tri and tetra-substituted product formation. Notably, such “self-protecting” attitude enabled the straightforward conjugation of the fourth nitrogen in the cyclen ring with a bioconjugable tether, starting from ethyl 7-bromoheptanoate. The length of the aliphatic arm was carefully selected to avoid possible intramolecular coordination of the carboxy group to the lanthanide ion. In addition, when 3 equivalents of **6** were employed, the regioisomeric mixture **7-bis** is obtained (see supplementary material). The latter will be subject of future studies as it allows to furtherly increase the degree of complexity of the final ligand.

Finally, we prepared **[Eu(Tris-B[h]IQ)]** (sodium salt) by basic hydrolytic cleavage of the ethyl esters groups present in **Tris-B[h]IQ**, followed by complexation in water with europium chloride hexahydrate. The 1:1 neutral (in its first coordination sphere) complex was purified by size exclusion chromatography and its identity and purity (98%) assessed by HPLC-MS (see supplementary material). Remarkably, the complex resulted stable in Tris-HCl pH = 7.4 for several weeks, as witnessed by the absence of dissociation product observed by HPLC-MS.

### 2.3. Photophysical Characterization

**Figure 3:**
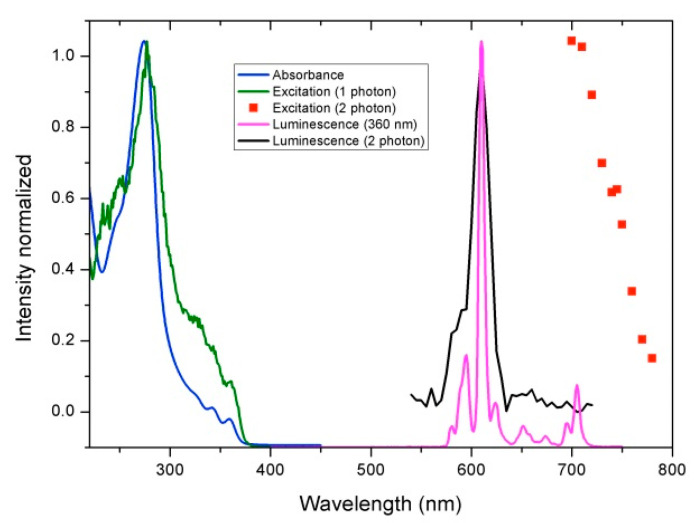
Absorption, excitation and one/two photon emission spectra of **Na[Eu(Tris-B[*h*]IQ)]** complex at pH = 7.4.

Photophysical characterization of **Na[Eu(Tris-B[h]IQ)]** in water at pH 7.4 is depicted in Figure 3. The absorption spectrum presents bands of remarkably different intensity: 245 nm (ε = 45,000 M^−1^cm^−1^), 270 nm (ε = 82,000 M^−1^cm^−1^), 325 nm (ε = 10,000 M^−1^cm^−1^), 340 nm (ε = 8000 M^−1^cm^−1^) and 360 nm (ε = 6000 M^−1^cm^−1^). The measured low-energy peaks at 325–360 nm confirm the values extrapolated from the TD-DFT calculation.

Albeit slightly overestimated, the predicted extinction coefficients fall in the experimental range and the higher ε of **Na[Eu(Tris-B[h]IQ)]** low-energy bands with respect to EuL1 (ε = 4000 M^−1^cm^−1^, ref. 20) is also confirmed. By exciting the complex at 360 nm, we collected the typical luminescence profile of the Eu^3+^ ion, [13] with ^5^D_0_→^7^F_J_ transitions at 580 nm (J = 0) nm, 55 nm (J = 1), 610 nm (J = 2), 650–675 nm (J = 3), 695–705 (J = 4). The splitting of the europium emission lines recalled the low symmetry of the system (*C_1_*), confirmed by the presence of the unique ^5^D_0_→^7^F_0_ band at 580 nm [13] Notably, the above photoluminescence pattern was also elicited by two-photon excitation (Figure 3). Although we were not able to measure the actual cross-sections, the intensity-normalized TPE spectrum indicated that **Na[Eu(Tris-B[h]IQ)]** can be excited at 750–780 nm, thus making this probe suitable for sensitization in the transparency NIR-I window of tissues [44].

The luminescence decay was found to be monoexponential with lifetime τ = 0.8 ± 0.05 ms (Supplementary Figure S29) in agreement with a unique Eu^3+^ species. According to theory [3], the radiative lifetime τ_r_ of Eu^3+^ can be calculated from the integrated total emission I_tot_ of the ^5^D_0_→^7^F_J_ transitions (J = 0–6) and the integrated emission I_MD_ of magnetic dipole transition ^5^D_0_→^7^F_1_, according to Equation (1):

(1) $$\;k_{r}=\frac{1}{\tau_{r}}\;=A_{MD,0}\cdot n^{3}\cdot \left(\frac{I_{tot}}{I_{MD}}\right)$$

where A_MD,0_ = 14.65 s^−1^ is the spontaneous emission probability of the ^5^D_0_→^7^F_1_ transition and n the refractive index of the medium. We found τ_r_ = 1.58 ms. The intrinsic quantum yield of the lanthanide ion $\Phi_{\mathrm{Eu}}^{\mathrm{Eu}}$ (i.e., the quantum yield upon direct 4f−4f excitation) is related to the luminescence and radiative lifetimes according to Equation (2): [3]

(2) $$\;\Phi_{\mathrm{Eu}}^{\mathrm{Eu}}=\tau /\tau_{r}\;$$

This leads to $\Phi_{\mathrm{Eu}}^{\mathrm{Eu}}$ = 0.51. For comparison, the intrinsic quantum yield of Eu^3+^-tris(dipicolinates) in water at pH = 7.4 accounts to 0.41 [45], whereas naphtyridine-based complexes were recently reported to own $\Phi_{\mathrm{Eu}}^{\mathrm{Eu}}$ = 0.18–0.33 [18]. The high intrinsic quantum yield and its associated significant reduction of non-radiative deactivation of excited Eu^3+^ witnesses the strong protective action of the cyclen scaffold of Tris-B[h]IQ towards the metal center. The photoluminescence yield of the complex upon ligand excitation ($\Phi_{\mathrm{Eu}}^{L}$) is simply the product of $\Phi_{\mathrm{Eu}}^{\mathrm{Eu}}$ and the sensitization efficiency η_sens_ of the antenna system Equation (3):

(3) $$\;\;\Phi_{\mathrm{Eu}}^{L}=\eta_{sens}\cdot \Phi_{\mathrm{Eu}}^{\mathrm{Eu}}$$

Rather surprisingly, we found out η_sens_ = 0.012 ± 0.0002 from the experimentally measured value $\Phi_{\mathrm{Eu}}^{L}$ = 6.1*10^−3^ ± 0.3*10^−3^ through equation 3. This finding is at odds with the energies of the major phosphorescence bands of benzoisoquinoline (21,650 and 21,370 cm^−1^), which fall in the region of highest sensitization of Eu^3+^ by triplet states [14]. Additionally, no quenching effect of water molecules can be invoked, since both the monoexponential lifetime decay and the unique ^5^D_0_→^7^F_0_ band at 580 nm indicate that no water molecules are present in the first coordination sphere of the complex. Two mutually non-excluding hypotheses may hold: i) for some reason the phosphorescence band of **Tris-B[*h*]IQ** deviates from the behavior of benzoisoquinoline, and/or ii) the intersystem crossing of the ligand is not efficient. The triplet state features of **Tris-B[*h*]IQ** will be the subject of a forthcoming study, which targets the structural manipulation of the ligand to raise the luminescence emission of the full complex without losing the valuable properties of **Na[Eu(Tris-B[*h*]IQ)]** here reported.

### 2.4. Structured Illumination Imaging in the Biological Context

We finally put to the test the use of the complex as imaging probe in indirect immunofluorescence (IIF) of biomolecules in cells. To do this, the carboxylic tether of **Na[Eu(Tris-B[h]IQ)]** was first activated to bioconjugation by sulfo-N-hydroxy succinimide, and then attached to a secondary donkey anti-rabbit antibody (**Ab-Eu**). After purification by dialysis, spectrophotometrically we measured a labelling degree of 2.8 metal centers per single molecule of **Ab-Eu**.

The luminescent secondary antibody was applied in an immunostaining procedure established by us that targets the nuclear protein BMI1 in cultured cells [46]. BMI1 is a member of the polycomb repressor complex 1 (PRC1), one of the most relevant epigenetic modulators of transcription in metazoan [47]. Recently, BMI1 was identified as relevant biochemical crossroad in several kinds of cancers [48], and his role as theranostic target has been proposed [49]. Accordingly, the immunostaining was carried out in A549 cells, a model line of human non-small cell lung cancer (NSCLC) that reportedly overexpresses BMI1 [50].

The immunofluorescence imaging was carried out by an epifluorescence microscope interfaced with a linear structured illumination (SIM) module [29]. Notably, this system allows an effective excitation of the Ln-complex around 360 nm by a conventional mercury lamp (or power LED) source, as well as 3D optical sectioning of the sample, strongly increasing the imaging contrast. The 3D sectioning capability of the system is similar to a confocal laser scanning microscope [51], although the latter comes seldom supplied with excitation laser sources below 400 nm and therefore requires adequate customization to use luminescent Ln-bioprobes by single photon excitation. Additionally, the SIM system is camera-based and does not require scanning of the sample, thus being much more tailored to μs-lifetime probes and time-gated experiments [10]. It is worth noting that SIM is emerging as sensitive and inexpensive technique for diagnostic imaging of tissues in biomedicine [52].

**Figure 4:**
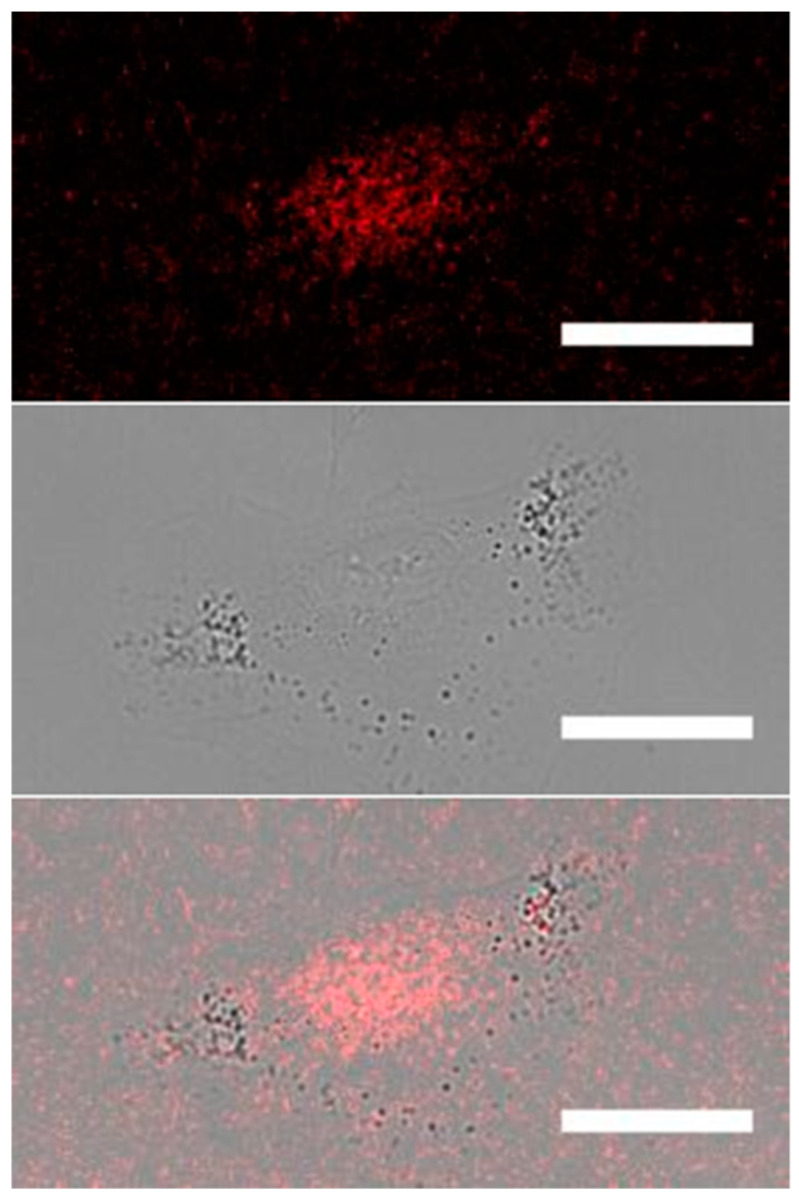
Indirect immunofluorescence of BMI1 in A549 cells by using **Ab**–**Eu** (see text). Top panel: luminescence image collected by a structured illumination microscopy system; middle panel: transmission image; bottom panel: overlay of luminescence and transmission. Scale bar: 20 μm.

Figure 4 displays an A549 cell immunostained by a commercial rabbit anti-BMI1 primary antibody followed by Ab-Eu. Emission was collected at λ > 575 nm. For each cell, the predominant nuclear localization of BMI1 was correctly observed. In spite of the cytoplasmatic and mitochondrial localization of UV-excitable endogenous fluorophores such as NADH and FAD, we found negligible emission in non-nuclear regions of the cell, likely on account of the large spectral separation between the lanthanide luminescence and the excited autofluorescence.

## 3. Materials and Methods

All solvents and chemicals were purchased from Sigma-Aldrich and used without further purification. Silica gel 60 F254 sheets (Merck, Darmstadt, Germany) were used in analytical thin-layer chromatography (TLC). LC chromatographic separation were performed on an RfCombiflash (Teledyne ISCO, Lincoln, NE, USA) preparative purification system. Evaporation was performed in vacuo (rotating evaporator). Sodium sulfate was used as drying agent. 1D and 2D-NMR were recorded with a Bruker Avance III 300 spectrometer (Bruker, Billerica, MA, USA), using the indicated deuterated solvents. Chemical shifts are given in parts per million (ppm) (δ relative to residual solvent peak for 1H and 13C). Purity and MS spectra of synthesized compounds were determined using a Shimadzu Nexera UHPC (Shimadzu, Nakagyo-ku, Kyoto, Japan) equipped with a diode array detector and interfaced with an ABSciex API 3200 QTRAP mass spectrometer (AB Sciex LLC, Framingham, MA, USA), using the parameters specified below: LC conditions: column: Phenomenex Proteo column (4.6 × 250 mm) (Phenomenex, Torrance, CA, USA) Mobile phases: water/TFA 100/0.01 *v*/*v* and Acetonitrile/Water/TFA 95/100/0.01 *v*/*v*. MS conditions: Ionization mode: ESI. Curtain gas 10 mL/min; Ion spray voltage: 5500 V; Temperature: not used; Declustering potential: 75 V; Entrance potential: 10 V; Collision energy: 50 eV; Collision energy potential: 43 V.

Retention times (HPLC, tR) are given in minutes. Compound HPLC purity was evaluated at 254 and 220 nm.

### 3.1. Synthetic Procedures and Characterization

Synthesis and Characterization of Compound **1**. In a two necks round bottom balloon, under nitrogen atmosphere, 10 g of 2-(bromomethyl)naphthalene (45 mmol, 1 eq) were added and dissolved in 150 mL of dry acetonitrile. Then, 9.8 g of diethylacetoaminomalonate (45 mmol, 1 eq), 12.5 g of anhydrous K_2_CO^3^ (90 mmol, 2 eq), 7.5 g of KI (45 mmol, 1 eq) were respectively added. The suspension was refluxed under nitrogen atmosphere for 12 h. In time the suspension turned pale yellow. Then the reaction was cooled down, filtered and the solvent evaporated and the solid dried. The crude compound was purified by liquid chromatography (80 g SiO_2_ column, solid state sampling, cyclohexane/ethyl acetate gradient elution) to afford 12.1 g of compound **1** as an off white solid (Yield = 76%). ^1^H-NMR (300 MHz, CDCl_3_) δ (ppm) = 7.80 (m, 1H), 7.74 (d, ^3^*J* = 8 Hz, 1H), 7.46 (m, 3H), 7.10 (d, ^3^*J* = 8 Hz, 1H), 6.53 (s, 1H), 4.30 (q, ^3^*J* = 7 Hz, 4H), 3.82 (s, 2H), 2.04 (s, 3H), 1.32 (t, ^3^*J* = 7 Hz, 6H). ^13^C-NMR (75 MHz, CDCl3) δ (ppm) = 169.2, 167.5, 133.3, 132.8, 132.5, 128.9, 127.9, 127.8, 127.6, 127.5, 126.1, 125.8, 67.4, 62.7, 37.9, 23.1, 14.1.

Synthesis and Characterization of Compound **2**. In a two-neck round bottom balloon, under nitrogen atmosphere, 6 g of **1** (17 mmol) were added and dissolved in 200 mL of freshly distilled phosphorus (V) oxychloride. The suspension was refluxed under nitrogen atmosphere for 1h. Then, the reaction was cooled down and the solvent distilled under reduced pressure. The residual solid was dissolved in dichloromethane and carefully extracted with a saturated solution of NaHCO_3_ and water until neutrality, then washed with brine. The organic phase was dried with anhydrous Na_2_SO_4_, the solution filtered, and the solvent evaporated. The crude compound was purified by liquid chromatography (80 g SiO_2_ column, solid state sampling, cyclohexane/ethyl acetate gradient elution) to afford 3.6 g of compound **2** as a pale-yellow solid (Yield = 63%). ^1^H-NMR (300 MHz, CDCl^3^) δ (ppm) = 8.21 (d, ^3^*J* = 8.3 Hz, 1H), 7.87 (d, ^3^*J* = 8.3 Hz, 1H), 7.84 (d, ^3^*J* = 7.7 Hz, 1H), 7.56 (t, ^3^*J* = 8.3 Hz, 1H), 7.49 (t, ^3^*J* = 7.2 Hz, 1H), 7.36 (d, ^3^*J* = 8.3 Hz, 1H), 4.16 (q, ^3^*J* = 7.1 Hz, 4H), 3.44 (s, 2H), 2.79 (s, 3H), 1.12 (t, ^3^*J* = 7.1 Hz, 6H). ^13^C-NMR (75 MHz, CDCl^3^) δ (ppm) = 169.2, 132.5, 129.0, 127.0, 125.7, 125.5, 125.4, 62.3, 33.8, 28.1, 13.8. The isomer isolated is the benzo[*h*]isoquinoline-like; the assignment of each NMR resonances has been done by HSQC and HMBC maps and the structure checked by ROESY (see supplementary material).

Synthesis and Characterization of Compound **3**. In a two-neck round bottom balloon, 3 g of compound **2** (8.8 mmol, 1 eq) were added under nitrogen atmosphere. Then, 100 mL of anhydrous pyridine and 4 g of sodium iodide (27 mmol, 3 eq) were respectively added. The reaction was refluxed overnight. Then, the solution was cooled down and the pyridine distilled away under reduced pressure. The residual solid was suspended in chloroform and washed with water and brine; the organic phase was dried with anhydrous Na_2_SO_4_, filtered and the solvent evaporated. The crude compound was purified by liquid chromatography (40 g SiO^2^ column, solid state sampling, cyclohexane/ethyl acetate gradient elution) to afford 920 mg of compound **3** as a pale-yellow solid (Yield = 40%). ^1^H-NMR (300 MHz, CDCl^3^) δ (ppm) = 8.97 (d, ^3^*J* = 8.3 Hz, 1H), 8.47 (s, 1H), 8.00 (d, ^3^*J* = 0 7.7 Hz, 1H), 7.99 (d, ^3^*J* = 8.4 Hz, 1H), 7.80–7.70 (m, 3H), 4.55 (q, ^3^*J* = 7 Hz, 2H), 3.46 (s, 3H), 1.50 (t, ^3^*J* = 7 Hz, 3H). ^13^C-NMR (75 MHz, CDCl_3_) δ (ppm) = 165.7, 157.7, 141.3, 137.9, 134.4, 132.3, 130.0, 129.3, 127.5, 127.4, 127.4, 126.3, 122.7, 61.9, 30.9, 14.4.

Synthesis and Characterization of Compound **4**. In a one-neck round bottom balloon, 920 mg of compound **3** (3.5 mmol, 1 eq) were dissolved in 50 mL of dioxane and 770 mg of selenium dioxide (7 mmol, 2 eq) were added. The reaction was refluxed for 1 h and then cooled down, filtered and the dioxane evaporated. The crude solid was purified by liquid chromatography (40 g SiO_2_ column, solid state sampling, cyclohexane/ethyl acetate gradient elution) to afford 600 mg of compound **4** as a pale-yellow solid (Yield = 62%). ^1^H-NMR (300 MHz, CDCl_3_) δ (ppm) = 10.69 (s, 1H), 8.70 (s, 1H), 8.58, (d, ^3^*J* = 8.1 Hz, 1H), 8.09 (d, ^3^*J* = 8.8 Hz, 1H), 8.00 (d, ^3^*J* = 7.6 Hz, ^4^*J* = 1.3 Hz, 1H), 7.85 (d, ^3^*J* = 8.8 Hz, 1H), 7.78 (t, ^3^*J* = 7.2 Hz, ^4^*J* = 1.3 Hz, 1H), 7.72 (t, ^3^*J* = 8 Hz, ^4^*J* = 1.5 Hz, 1H), 4.58 (q, ^3^*J* = 7.1 Hz, 2H), 1.50 (t, ^3^*J* = 7 Hz, 3H). ^13^C-NMR (75 MHz, CDCl_3_) δ (ppm) = 164.8, 152.8, 142.3, 138.5, 134.3, 133.5, 129.4, 129.0, 128.5, 127.6, 127.2, 126.6, 126.5, 125.2, 62.2, 14.4.

Synthesis and Characterization of Compound **5**. In a two-neck round bottom balloon, 600 mg of **4** (2.2 mmol, 1 eq) were dissolved in 120 mL of dry ethanol and 30 mL of anhydrous chloroform under nitrogen atmosphere. The solution was cooled down at −20 °C with an ice/CaCl_2_ bath and 41 mg of NaBH_4_ (1.1 mmol, 0.5 eq) were added at once and the solution was kept at −20 °C for 30 min. Then, the reaction was quenched with 20 mL of HCl 1M; the separated organic layer was washed with a saturated solution of NaHCO_3_, water until neutrality and finally brine. The organic phase was dried with anhydrous Na_2_SO_4_, filtered and the solvent evaporated to afford 520 mg of pale yellow solid **5**, which was used without any further purification (Yield = 86%). ^1^H-NMR (300 MHz, CDCl_3_) δ (ppm) = 8.59 (s, 1H), 8.45 (d, ^3^*J* = 8.6 Hz, 1H), 8.11 (d, ^3^*J* = 8.8 Hz, 1H), 8.07 (d, ^3^*J* = 7.8 Hz, ^4^*J* = 1.3 Hz, 1H), 7.90 (d, ^3^*J* = 8.8 Hz, 1H), 7.87 (t, ^3^*J* = 6.7 Hz, ^4^*J* = 1.7 Hz, 1H), 7.80 (t, ^3^*J* = 7.4 Hz, 1H), 5.57 (s, 2H), 4.55 (q, ^3^*J* = 7.1 Hz, 2H), 1.51 (t, ^3^*J* = 7.1 Hz, 3H). ^13^C-NMR (75 MHz, CDCl_3_) δ (ppm) = 164.6, 157.6, 138.5, 134.3, 133.4, 129.7, 129.0, 128.5, 128.2, 127.2, 126.2, 125.9, 123.9, 65.6, 62.0, 14.4.

Synthesis and Characterization of Compound **6**. In a one neck round bottom balloon, 100 mg of **5** (0.36 mmol, 1 eq) were dissolved in 50 mL of chloroform. Then, 200 µL of triethylamine (1.08 mmol, 3 eq) and 45 µL of mesyl chloride (0.54 mmol, 1.5 eq) were respectively added. The reaction was kept at room temperature for 30 min, then 20 mL of a saturated solution of NaHCO_3_ was added and the organic phase was extracted with water until neutrality. The solution was dried with anhydrous Na_2_SO_4_, filtered and the solvent evaporated to afford 140 mg of compound **6** as yellow solid, which was used without any further purification (Yield = quantitative). ^1^H-NMR (300 MHz, CDCl_3_) δ (ppm) = 8.62 (d, ^3^*J* = 8.3 Hz, 1H), 8.60 (s, 1H), 8.06 (d, ^3^*J* = 8.6 Hz, 1H), 8.04 (d, ^3^*J* = 7.4 Hz, ^4^*J* = 1.6 Hz, 1H), 7.86 (t, ^3^*J* = 7.1 Hz, ^4^*J* = 1.6 Hz, 1H), 7.83 (d, ^3^*J* = 8.6 Hz, 1H), 7.79 (t, ^3^*J* = 7.3Hz, ^4^*J* = 1 Hz, 1H), 6.15 (s, 2H), 4.53 (q, ^3^*J* = 7.2 Hz, 2H), 3.74 (s, 3H), 1.49 (t, ^3^*J* = 7.2 Hz, 3H). ^13^C-NMR (75 MHz, CDCl_3_) δ (ppm) = 165.0, 151.4, 141.8, 138.4, 134.3, 133.1, 129.5, 128.6, 128.4, 128.1, 127.4, 127.2, 125.8, 124.5, 73.4, 61.9, 39.2, 14.4.

Synthesis and Characterization of Compound **7-Tris**. In a two-neck round bottom balloon, 56 mg of cyclen tetrahydrochloride (0.18 mmol, 1 eq) were dissolved under nitrogen atmosphere in 100 mL of dry acetonitrile. Then, were respectively added 250 µL of N,N-diisopropylethylamine (1.41 mmol, 8 eq) and 250 mg of mesylate **6** (0.72 mmol, 4 eq). The reaction was refluxed under nitrogen atmosphere for 3 days. Then the solution was cooled down and the solvent evaporated. The crude product was purified by liquid chromatography (RP-C_18_ 26 g column, water with 0.1% TFA-acetonitrile gradient elution, on column sampling) to afford 70 mg of compound **7-Tris** as a brown glassy solid (Yield = 41%). ^1^H-NMR (300 MHz, CDCl_3_) δ (ppm) = 8.43–7.63 (m, 21H), 5.72–3.33 (m, 29H), 1.43–1.12 (m, 9H). ^13^C-NMR (75 MHz, CDCl_3_) δ (ppm) = 165.1, 164.6, 161.2, 160.7, 43.4, 140.2, 138.8, 138.0, 134.5, 134.4, 132.6, 129.8, 129.7, 129.3, 128.6, 127.8, 127.6, 127.3, 127.1, 126.9, 126.3, 125.2, 123.1, 118.0, 114.1, 61.5, 53.3, 49.1, 43.1, 29.7, 14.2, 13.9. Purity 98%, assessed by HPLC-MS (Jupiter Proteo analytical column), ret. time 10.3 min, mass spectrum (*m*/*z*), 963 [M + H]^+^, 482 [M + 2H]^2+^.

Synthesis and Characterization of **Tris-B[h]IQ**. In a two-neck round bottom balloon, 50 mg of compound **7-Tris** (0.05 mmol, 1 eq) were dissolved in 30 mL of dry acetonitrile under nitrogen atmosphere. Then, were respectively added 600 µl of N,N-diisopropylethylamine (3 mmol, 60 eq), 600 µL of ethyl 7-bromoheptanoate (3 mmol, 60 eq) and 450 mg of sodium iodide (3 mmol, 60 eq). The reaction was refluxed under nitrogen atmosphere for 3 days. Then, the solution was cooled down and the solvent evaporated. The residual solid was suspended in 50 mL of dichloromethane and the organic phase was washed with water and brine, dried with anhydrous sodium sulfate, filtered and the solvent evaporated. The crude product was purified by preparative HPLC (Proteo Semiprep, water with 0.01% TFA-acetonitrile with 5% water and 0.01% TFA, solvent gradient from 40% acetonitrile-based solvent to 100% in 7 min of a 12 min total run) to afford 30 mg of compound **Tris-B[h]IQ** as a brown glassy solid (Yield = 52%, purity 97% assessed by HPLC). ^1^H-NMR (300 MHz, CDCl_3_) δ (ppm) = 8.49–7.71 (m, 21H), 5.51–3.40 (m, 30H), 2.30–1.11 (m, 21H). ^13^C-NMR (75 MHz, CDCl_3_) δ (ppm) = 173.5, 164.9, 155.0, 143.5, 140.5, 139.7, 138.0, 134.4, 134.4, 132.7, 129.6, 129.5, 128.8, 128.4, 128.2, 127.9, 127.6, 127.2, 126.0, 125.7, 124.7, 122.9, 118.3, 114.5, 62.4, 61.7, 60.2, 52.7, 50.1, 48.2, 40.9, 33.8, 28.3, 26.2, 24.3, 14.1, 14.0. Purity assessed by HPLC-MS (Jupiter Proteo analytical column), ret. time 10.7 min, mass spectrum (*m*/*z*), 1118 [M + H]^+^, 559.5 [M + 2H]^2+^.

Synthesis and Characterization of **Na[Eu(Tris-B[h]IQ)]**. In a one-neck round bottom balloon, 10 mg of **Tris-B[h]IQ** (9µmol, 1 eq) were dissolved in 10 mL of a 1:1 mixture of water and ethanol. Then, 200µl of NaOH 1M (0.18 mmol, 20 eq) were added and the solution was refluxed until complete saponification (HPLC-MS followed). The ethanol was then evaporated under reduced pressure and the pH adjusted to 7 with HCl 1M. Finally 3.6 mg of EuCl_3_*6H_2_O (9.9 µmol, 1.1 eq) were added and the final pH checked to be around 7. The solution was then concentrated to ca. 1 mL and the europium complex was purified (to get rid mainly of the salts) by Size Exclusion chromatography (LH−20, using water as eluent) to afford 4.6 mg of **Na[Eu(Tris-B[h]IQ)]** complex as pale yellow glassy solid (Yield = 46%). Purity 98%, assessed by HPLC-MS (Jupiter Proteo analytical column, 20 mM ammonium acetate/acetonitrile with a gradient 0 to 100% acetonitrile in 18 min): ret. time 7.2 min, mass spectrum (*m*/*z*) 1155 [M + H]^+^, 577 [M + 2H]^2+^.

### 3.2. Photophysical Characterization of Na[Eu(Tris-B[h]IQ)]

Absorption and fluorescence spectra were recorded in cuvettes with 1 cm optical path (Hellma, Müllheim, Germany) at 23 °C on a Jasco V550 spectrophotometer (Jasco, Easton, MD, USA) and a Cary Eclipse spectrofluorometer (Varian, Palo Alto, CA, USA), respectively. A stock solution of Na[Eu(Tris-B[h]IQ)] in DMSO (2 mM, as determined by ICP-MS), was diluted 1:200 (to 10 µM) in Tris-HCl buffer pH 7.4, and then absorbance and fluorescence were measured. The actual complex concentration was used to calculate the molar absorption spectrum of the complex (see Figure S28 in supplementary material). The luminescence lifetime τ of the complex was measured by the phosphorescence plugin of the Cary-Eclipse spectrofluorometer. The luminescence decay was fitted to a monoexponential decay to recover τ (see Supplementary Figure S29). For the determination of the quantum yield of the complex $(\Phi_{\mathrm{Eu}}^{L}$), we measured the absorbance and fluorescence of several solutions of quinine sulfate in 0.5 M H_2_SO_4_ ($\Phi_{\mathrm{QS}}=0.56)$ and of the Eu complex in Tris-HCl pH 7.4 (see Supplementary Figure S30). Note that solution absorbances were in all cases below 0.1.

### 3.3. Conjugation of Na[Eu(Tris-B[h]IQ)] with Secondary IgG Donkey Anti Rabbit Antibody (Ab-Eu)

Complex **Na[Eu(Tris-B[h]IQ)]** was incubated at pH = 6 with an excess (1000 fold mol/mol) of EDC and sulfo-NHS. Reaction was stirred overnight at 25 degrees and the obtained product was added (1000:1 molar ratio between the complex and the antibody) to an antibody solution (AffiniPure Donkey anti-Rabbit IgG H+L, Jackson ImmunoResearch) buffered at pH 7.1 and the resulting solution was stirred overnight at 4 °C, then dialyzed on centrifugal concentrators with a 100 kDa cutoff. Concentrated solution was repeatedly washed with PBS until no fluorophore was detectable in the filtrate. The conjugated antibody **Ab-Eu** was stored at 4 degrees until use and characterized by UV-VIS and fluorescence spectroscopy. Ratio between absorbance at 280 and 360 nm allowed determining a fluorophore/antibody ratio of 2.8. The conjugation of Eu complex to the antibody was further confirmed by evaluating its retention time in GPC and the presence of Europium in the eluted fraction at the expected retention time for the antibody. A sample of labelled antibody (50 uL) was eluted and purified by size exclusion chromatography on a SuperdexTM 200 Increase 10/300 GL (V0 = 8.05 mL) equilibrated with 10 mM phosphate buffer, pH 7.6, containing 250 mM sodium chloride and previously calibrated using a mixture of Ferritin (440 kDa), Aldolase (158 kDa) and Cytochrome C (12.4 kDa). The flow rate through the column was kept at 0.750 mL/min. Retention time of the only peak absorbing at 280 nm is fully coherent with the expected molecular weight of the antibody (Supplementary Figure S32). The eluted fraction containing the peak of interest was collected and digested in HCl 6M at 95 °C for 12 h. After digestion the sample was dried, reconstituted in HNO_3_ 2% in water for trace analysis and evaluated for Eu content by ICP-MS, comparing the result with a standard calibration curve. Results confirmed the presence of europium in the fraction containing the antibody ([Eu] = 3.5 nM in the analysed sample).

### 3.4. Cell Cultures

Adenocarcinoma human alveolar basal epithelial cells (A549) were grown in Roswell Park Memorial Institute (RPMI) 1640 medium (RPMI 1640, Invitrogen, Carlsbad, CA, USA) supplemented with 10% Fetal Bovine Serum (FBS), glutamine (2mM), 100 U/mL penicillin and 100 mg/mL streptomycin (Invitrogen). Cells were maintained at 37 °C in a humidified 5% CO_2_ atmosphere. For live imaging, 6–7 × 104 cells were plated on a 35-mm glass bottom dish (Willco-dish HBST-3512/1.5-0.005) 24–48 h before performing the immunofluorescence experiments.

### 3.5. Immunostaining

A549 cells were washed with phosphate buffer saline 1x (PBS, 3 times) and then fixed with paraformaldehyde (2% in PBS) for 15 min. After washing with PBS (3 times), cells were permeabilized with 0.1% Triton X−100 made in PBS, for 15 min. Cells were then washed with PBS (3 times), 0.5% Bovine Serum Albumin in PBS (PBB) 4 times, and exposed for 40 min to 2% Bovine Serum Albumin in PBS (BSA 2%). After washing with PBB (4 times), cells were incubated with Rabbit anti-human BMI1 monoclonal antibody (Cell Signaling Technologies #6964S; 1:600 dilution in PBB) for 1h at room temperature (RT) and additional 1.5 h at 4 °C. Cells were washed with PBB (4 times), and incubated with the secondary antibody **Ab-Eu** at 1:100 dilution in PBB) for 1h at RT in dark. Next, cells were washed with PBB (4 times) and PBS (3 times). Cells were then maintained in PBS at 4 °C before imaging.

### 3.6. Fluorescence Microscopy Imaging

Fluorescence imaging was carried out by a Nikon TI2-E (Nikon Italia, Florence, Italy) inverted microscope interfaced with a ViCo−2 videoconfocal (Biomedica Mangoni, Pisa, Italy) structured illumination module. More specifically, the ViCo slider module was inserted into the field diaphragm of the microscope, so that the rectangular-shaped gratings could project on the sample a modulated excitation pattern. The microscope was also interfaced with an HG motorized Intensilight (Nikon Italia, Florence, Italy) as illumination source and a DS-Qi2 Mono Digital Microscope. Ab-Eu luminescence was excited by using a 360/20 filter (Semrock, USA). A TRITC-B-000 dichroic mirror (Semrock, USA) was used to separate out excitation from emission light. No emission filter was placed in the microscope, since the dichroic mirror allowed light with λ > 570 nm to reach the camera. Imaging was carried out by using a 63x CFI Plan Apochromat Lambda oil objective (Nikon Italia, Florence, Italy). Camera integration time was set in the range 0.5–1 s. Images were collected by NIS Element Ar (Nikon Italia, Florence, Italy) and then elaborated by ImageJ 1.51 (NIH, Bethesda, MD, USA).

### 3.7. Computational Methods and Additional Results

Geometry optimization of the analyzed structures was carried out at the density functional theory (DFT) level, using B3LYP as exchange and correlation (xc) potential, and a 6–31G(d) basis set, unless otherwise specified. For computational convenience the bioconjugable tether was replaced by a methyl group (see Figure S33). For the lanthanide ions we used the large-core quasi-relativistic effective core potential (ECP) from Stuttgart [53,54] and the related [5s4p3d]-GTO valence basis set. For comparison we also performed calculations employing the LANL2DZ (Los Alamos effective core potential plus double zeta) pseudopotential and basis set combination, substituting Eu with La, for which the pseudopotential is available [55]. The substitution with La has negligible effects on the lowest excitations (see Supplementary Figure S35). The basis set data were taken from the basis-set exchange website www.basissetexchange.org [56] and from www.tc.uni-koeln.de/PP/index.en.html. Excitation-energy calculations by time-dependent density functional theory (TD-DFT) where performed using the CAM-B3LYP exchange and correlation (xc) potential [57] and the same pseudopotential and basis sets reported above, unless otherwise specified. Implicit solvent effects were included using the polarizable continuum model [58] in the C-PCM variant [59] and the UAHF solute cavity [60]. The extinction coefficient ε(ν) was calculated as a sum of Gaussian function centered on the (singlet) excitation energy and with a variance σ = 0.1 eV (806.573 cm^−1^) and height determined by the oscillator strength $f_{i}$ of the excitation at wavenumber $\nu_{i}$ (in cm^−1^).

## 4. Conclusions

In conclusion, by a rational design strategy taking advantage of computational predictions, we synthesized a novel Eu^3+^ complex, **Na[Eu(Tris-B[*h*]IQ)]**, which possess strong stability in physiological water medium and bioconjugation capability. Our synthetic approach exploited the insertion of bulky, highly conjugated ligands that avoid the need for tedious protection/deprotection steps. This “self-protecting” approach is expected, in perspective, to enable the synthesis of new structures with more complex symmetry properties and ability to differentiate ligands. The complex resulted fairly soluble and stable in water at physiological pH.

This new complex represents an interesting starting platform for developing luminescent probes for bioimaging applications. In keeping with the computational calculations, the luminescence of Eu^3+^ could be achieved by excitation of the benzoisoquinoline antenna system at 365 nm, with a >20 nm red-shift if compared with a less conjugated isoquinoline ligand. The long luminescent decay time (800 μs) makes **Na[Eu(Tris-B[*h*]IQ)]** suitable for time-resolved experiments to gate off fast-decaying signals such as fluorescence. Notably, complex luminescence could be obtained by two-photon excitation at >700 nm, i.e., in the NIR-I spectral window of tissue bioimaging.

In spite of the low luminescent quantum yield, due to hampered sensitization efficiency of the ligand, we validated the use of the complex in indirect immunofluorescence imaging by targeting in cells BMI1, a nuclear protein working in the Polycomb Repressor Complex 1 and an established biomarker of some tumors. The use of a conventional epifluorescence microscope equipped with a linear structured illumination module disclosed a simple and inexpensive way to image confocally Ln-bioprobes by single photon excitation in the 350–400 nm window, where ordinary confocal systems have no excitation sources. Additionally, the camera-based SIM allows for parallelized detection of the luminescence from all the spatial points in the image over the long decay times of Ln complexes, a feature that is not possible for ordinary confocal systems characterized by fast raster-scanning mode of the sample. We should observe that the use of a bioconjugated lanthanide complex in such a relatively simple imaging setup is a step forward in the field of optical imaging of cells and tissue at high spatial resolution. Combined with the capability of organic lingands to modulate optical properties in response to protein binding events [22], our innovative setup could play an important role in signalling biorecognition events even in complex biological environments with minimal instrumental complexity.

## Data Availability

Data contained within the article and supplementary materials are available on request from the authors.

## Supplementary Materials

The following are available online. NMR/ESI-MS spectra of all the intermediates and final ligand and complex, luminescence lifetime spectra and and quantum yield plot, bioconjugation, cell cultures and immunostaining details and further computational methods.

## Sample Availability

Samples of the compounds are not available from the authors.
