# Supplementary material for: New 1,3-Disubstituted Benzo[h]Isoquinoline Cyclen-Based Ligand Platform: Synthesis, Eu3+ Multiphoton Sensitization and Imaging Applications
Source: Molecules. 2020 Dec 24;26(1):58. doi: 10.3390/molecules26010058 (PMC7795479; doi:10.3390/molecules26010058)
Supplement: Supplementary file 1 [file molecules-26-00058-s001.pdf]

Supporting information for "New 1,3-disubstituted benzo[h]isoquinoline cyclen-based ligand platform: synthesis, Eu3+ multi photons sensitization and imaging application"

Sebastiano Di Pietro, Dalila Iacopini, Barbara Storti, Riccardo Nifosì, Valeria Di Bussolo, Mauro Pineschi, Aldo Moscardini, Giovanni Signore and Ranieri Bizzarri

Contents

Material and methods 3

Synthetic procedures and characterization 4

Synthesis and characterization of compound 1 4

Synthesis and characterization of compound 2. 6

Figure S4. 7

Figure S5 7

Figure S6 8

Figure S7 8

Synthesis and characterization of compound 3. 9

Synthesis and characterization of compound 4. 11

Synthesis and characterization of compound 5 13

Synthesis and characterization of compound 6. 15

Synthesis and characterization of compound 7-Tris. 17

Synthesis and characterization of compound 7-Bis 19

Synthesis and characterization of Tris-B[h]IQ. 21

Synthesis and characterization of Na[Eu(Tris-B[h]IQ)]. 24

Photophysical characterization of Na[Eu(Tris-B[h]IQ)]. 25

Conjugation of Na[Eu(Tris-B[*h*]IQ)] with secondary IgG donkey anti rabbit antibody (Ab-Eu) 29

Cell cultures 32

Immunostaining 33

Fluorescence microscopy imaging 34
Computational methods and additional results 35
benzo[*h*]isoquinoline (B[*h*]IQ) 36
Eu/La(Tris-B[*h*]IQ) 38
References 40

Material and methods.

All solvents and chemicals were purchased from Sigma-Aldrich and used without further purification. Silica gel 60 F254 sheets (Merck) were used in analytical thin-layer chromatography (TLC). LC chromatographic separation were performed on an RfCombiflash preparative purification system. Evaporation was performed in vacuo (rotating evaporator). Sodium sulfate was used as drying agent

^{1}D and ^{2}D-NMR were recorded with a Bruker Avance III 300 spectrometer, using the indicated deuterated solvents. Chemical shifts are given in parts per million (ppm) (δ relative to residual solvent peak for ^{1}H and ^{13}C).

Purity and MS spectra of synthesized compounds were determined using a Shimadzu Nexera UHPC equipped with a diode array detector and interfaced with an ABSciex API 3200 QTRAP mass spectrometer, using the parameters specified below:

LC conditions: column: Phenomenex Proteo column (4.6x250 mm) Mobile phases: water/TFA 100/0.01 v/v and Acetonitrile/Water/TFA 95/100/0.01 v/v.

MS conditions: Ionization mode: ESI. Curtain gas 10 mL/min; Ion spray voltage: 5500 V; Temperature: not used; Declustering potential: 75 V; Entrance potential: 10 V; Collision energy: 50 eV; Collision energy potential: 43 V.

Retention times (HPLC, t_R) are given in minutes. Compound HPLC purity was evaluated at 254 and 220 nm.

Synthetic procedures and characterization.

Synthesis and characterization of compound 1. In a two necks round bottom balloon, under nitrogen atmosphere, 10 g of 2-(bromomethyl)naphthalene (45 mmol, 1 eq) were added and dissolved in 150 ml of dry acetonitrile. Then, 9.8 g of diethylacetoaminomalonate (45 mmol, 1eq), 12.5 g of anhydrous K_2CO_3 (90 mmol, 2 eq), 7.5 g of KI (45 mmol, 1 eq) were respectively added. The suspension was refluxed under nitrogen atmosphere for 12 h. In time the suspension turned pale yellow. Then the reaction was cooled down, filtered and the solvent evaporated and the solid dried. The crude compound was purified by liquid chromatography (80 g SiO_2 column, solid state sampling, cyclohexane/ethyl acetate gradient elution) to afford 12.1 g of compound **1** as an off white solid (Yield = 76%). ^{1}H-NMR (300 MHz, $CDCl_3$) δ (ppm) = 7.80 (m, 1H), 7.74 (d, ^3J = 8 Hz, 1H), 7.46 (m, 3H), 7.10 (d, ^3J = 8 Hz, 1H), 6.53 (s, 1H), 4.30 (q, ^3J = 7 Hz, 4H), 3.82 (s, 2H), 2.04 (s, 3H), 1.32 (t, ^3J = 7 Hz, 6H). ^{13}C-NMR (75 MHz, $CDCl_3$) δ (ppm) = 169.2, 167.5, 133.3, 132.8, 132.5, 128.9, 127.9, 127.8, 127.6, 127.5, 126.1, 125.8, 67.4, 62.7, 37.9, 23.1, 14.1.

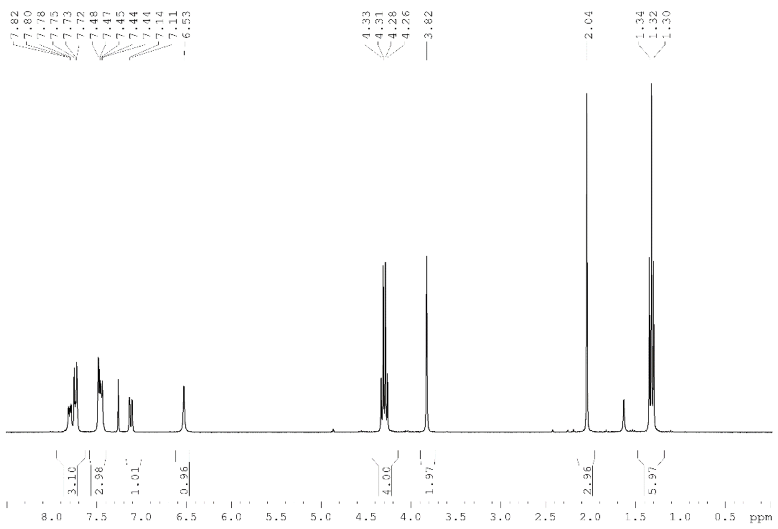

Figure S1. Compound 1 ^{1}H-NMR.

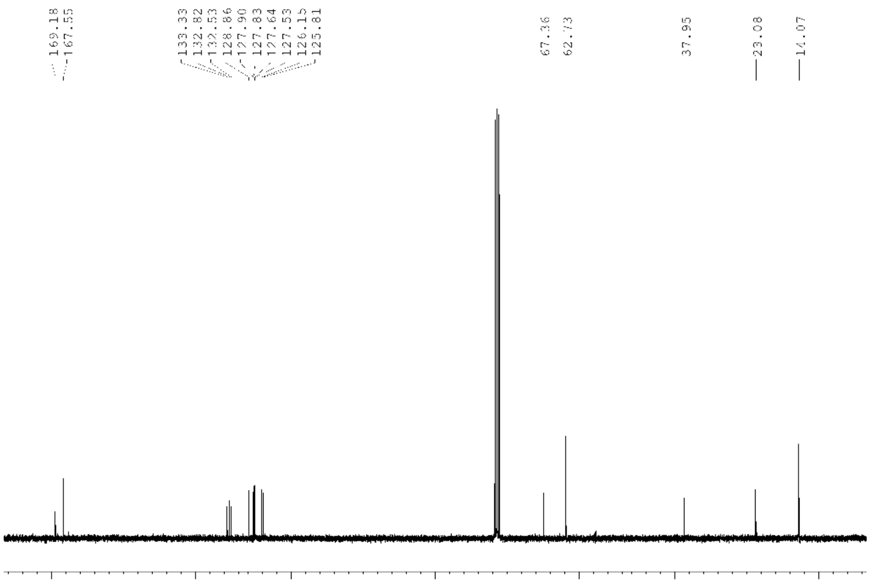

Figure S2. Compound 1 ^{13}C-NMR.

Synthesis and characterization of compound 2. In a two necks round bottom balloon, under nitrogen atmosphere, 6 g of **1** (17 mmol) were added and dissolved in 200 ml of freshly distilled phosphorus (V) oxychloride. The suspension was refluxed under nitrogen atmosphere for 1h. Then, the reaction was cooled down and the solvent distilled under reduced pressure. The residual solid was dissolved in dichloromethane and carefully extracted with a saturated solution of $NaHCO_3$ and water until neutrality, then washed with brine. The organic phase was dried with anhydrous Na_2SO_4, the solution filtered and the solvent evaporated. The crude compound was purified by liquid chromatography (80 g SiO_2 column, solid state sampling, cyclohexane/ethyl acetate gradient elution) to afford 3.6 g of compound **2** as a pale yellow solid (Yield = 63%). ^{1}H-NMR (300 MHz, $CDCl_3$) δ (ppm) = 8.21 (d, ^3J = 8.3 Hz ,1H), 7.87 (d, ^3J = 8.3 Hz, 1H), 7.84 (d, ^3J = 7.7 Hz, 1H), 7.56 (t, ^3J = 8.3 Hz, 1H), 7.49 (t, ^3J = 7.2 Hz, 1H), 7.36 (d, ^3J = 8.3 Hz ,1H), 4.16 (q, ^3J = 7.1 Hz ,4H), 3.44 (s, 2H), 2.79 (s, 3H), 1.12 (t, ^3J = 7.1 Hz, 6H). ^{13}C-NMR (75 MHz, $CDCl_3$) δ (ppm) = 169.2, 132.5, 129.0, 127.0, 125.7, 125.5, 125.4, 62.3, 33.8, 28.1, 13.8. The isomer isolated is the benzo[*h*]isoquinoline-like; the assignment of each NMR resonances has been done by HSQC and HMBC maps and the structure checked by ROESY.

Figure S3. Compound 2 full NMR assignment (grey arrows are ROESY cross peak signals).

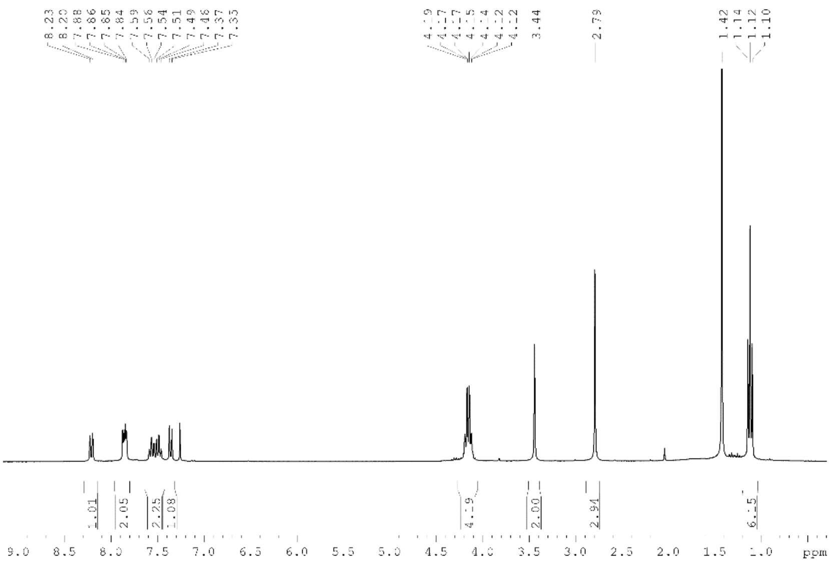

Figure S4. ^{1}**H-NMR.**

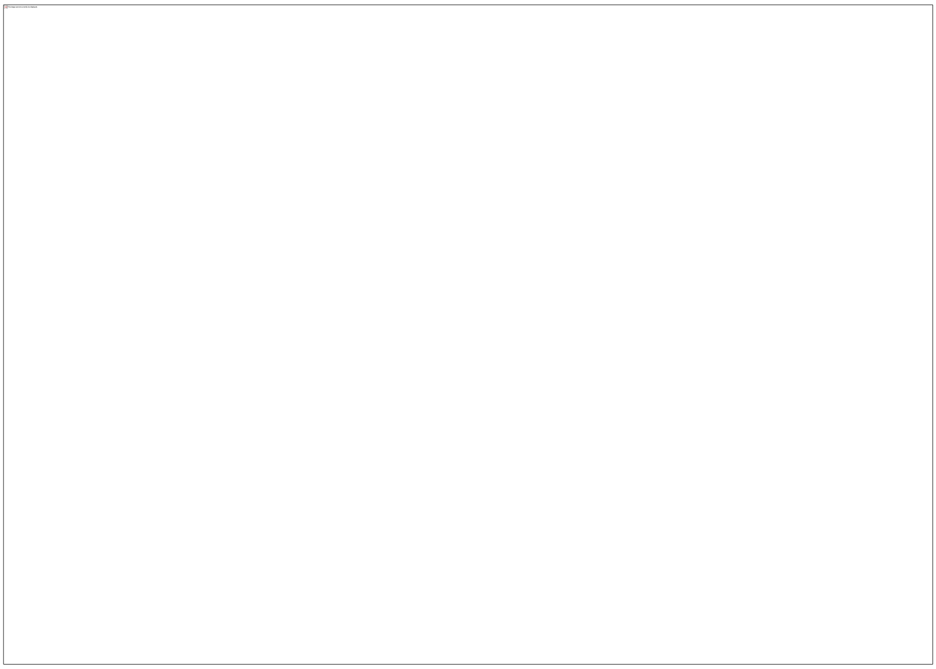

Figure S5 HSQC map.

This image cannot currently be displayed.

Figure S6 HMBC map.

This image cannot currently be displayed.

Figure S7 ROESY map.

Synthesis and characterization of compound 3. In a two necks round bottom balloon, 3 g of compound **2** (8.8 mmol, 1eq) were added under nitrogen atmosphere. Then, 100 ml of anhydrous pyridine and 4 g of sodium iodide (27 mmol, 3 eq) were respectively added. The reaction was refluxed overnight. Then, the solution was cooled down and the pyridine distilled away under reduced pressure. The residual solid was suspended in chloroform and washed with water and brine; the organic phase was dried with anhydrous Na_2SO_4, filtered and the solvent evaporated. The crude compound was purified by liquid chromatography (40 g SiO_2 column, solid state sampling, cyclohexane/ethyl acetate gradient elution) to afford 920 mg of compound **3** as a pale yellow solid (Yield = 40%). ^{1}H-NMR (300 MHz, $CDCl_3$) δ (ppm) = 8.97 (d, ^3J = 8.3 Hz, 1H), 8.47 (s, 1H), 8.00 (d, ^3J 0 7.7 Hz, 1H), 7.99 (d, ^3J = 8.4 Hz, 1H), 7.80-7.70 (m, 3H), 4.55 (q, ^3J = 7 Hz, 2H), 3.46 (s, 3H), 1.50 (t, ^3J = 7Hz, 3H). ^{13}C-NMR (75 MHz, $CDCl_3$) δ (ppm) = 165.7, 157.7, 141.3, 137.9, 134.4, 132.3, 130.0, 129.3, 127.5, 127.4, 127.4, 126.3, 122.7, 61.9, 30.9, 14.4.

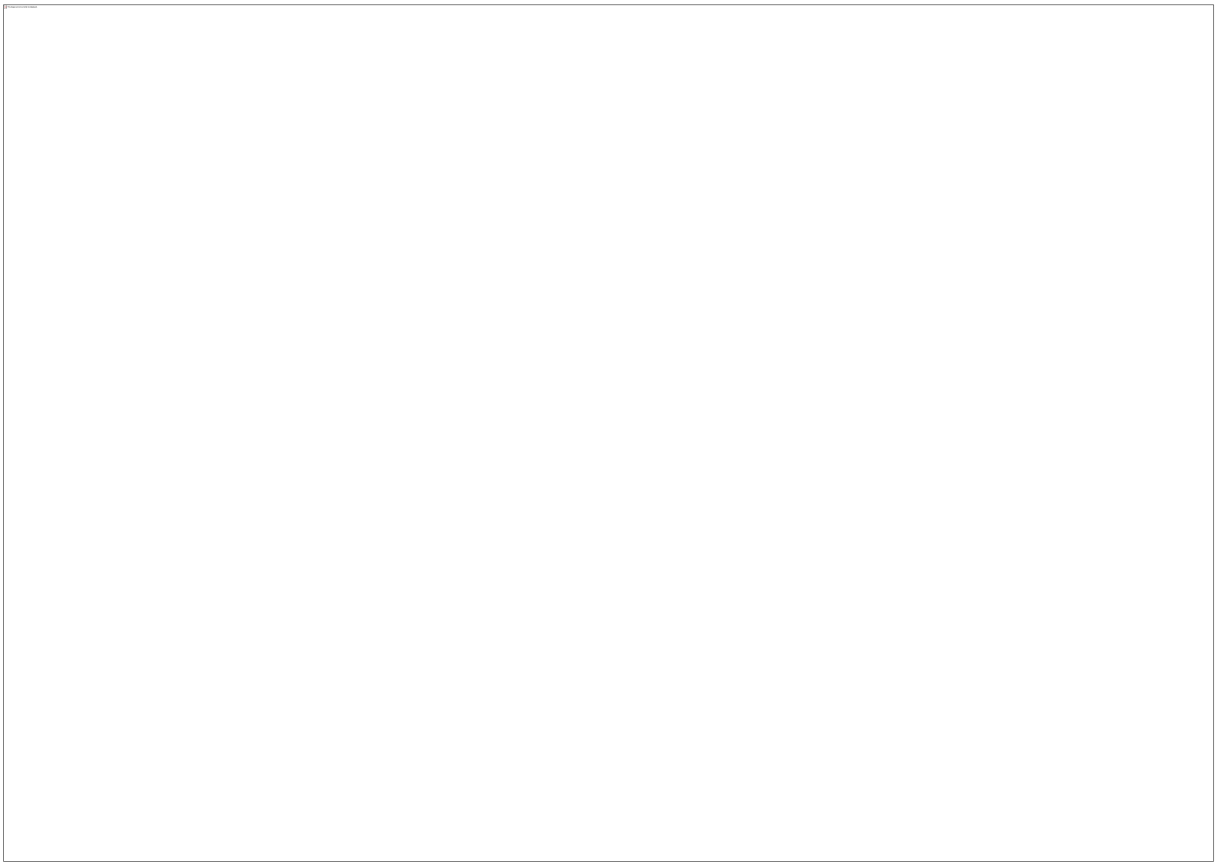

Figure S5. Compound 3 ^{1}H-NMR.

This image cannot currently be displayed.

Figure S6. Compound 3 ^{13}C-NMR.

Synthesis and characterization of compound 4. In a one neck round bottom balloon, 920 mg of compound **3** (3.5 Mmol, 1 eq) were dissolved in 50 ml of dioxane and 770 mg of selenium dioxide (7 Mmol, 2 eq) were added. The reaction was refluxed for 1 h and then cooled down, filtered and the dioxane evaporated. The crude solid was purified by liquid chromatography (40 g SiO_2 column, solid state sampling, cyclohexane/ethyl acetate gradient elution) to afford 600 mg of compound **4** as a pale yellow solid (Yield = 62%). ^{1}H-NMR (300 MHz, $CDCl_3$) δ (ppm) = 10.69 (s, 1H), 8.70 (s, 1H), 8.58, (d, ^3J = 8.1 Hz, 1H), 8.09 (d, ^3J = 8.8 Hz, 1H), 8.00 (d, ^3J = 7.6 Hz, ^4J = 1.3 Hz, 1H), 7.85 (d, ^3J = 8.8 Hz, 1H), 7.78 (t, ^3J = 7.2 Hz, ^4J = 1.3 Hz, 1H), 7.72 (t, ^3J = 8 Hz, ^4J = 1.5 Hz, 1H), 4.58 (q, ^3J = 7.1 Hz, 2H), 1.50 (t, ^3J = 7 Hz, 3H). ^{13}C-NMR (75 MHz, $CDCl_3$) δ (ppm) = 164.8, 152.8, 142.3, 138.5, 134.3, 133.5, 129.4, 129.0, 128.5, 127.6, 127.2, 126.6, 126.5, 125.2, 62.2, 14.4.

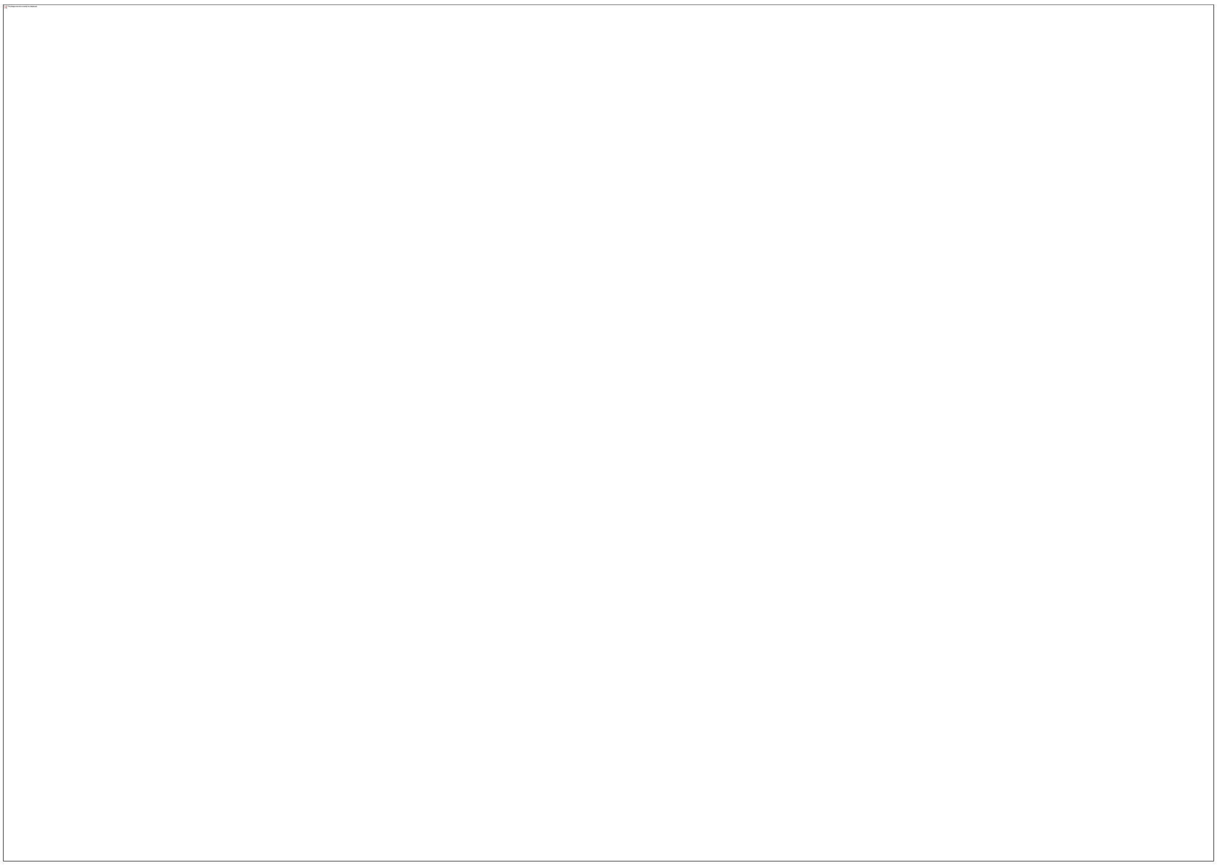

Figure S7. Compound 4 ^{1}H-NMR.

This image cannot currently be displayed.

Figure S8. Compound 4 ^{13}C-NMR.

Synthesis and characterization of compound 5. In a two necks round bottom balloon, 600 mg of **4** (2.2 mmol, 1 eq) were dissolved in 120 ml of dry ethanol and 30 ml of anhydrous chloroform under nitrogen atmosphere. The solution was cooled down at -20°C with an ice/$CaCl_2$ bath and 41 mg of $NaBH_4$ (1.1 mmol, 0.5 eq) were added at once and the solution was kept at -20°C for 30 minutes. Then, the reaction was quenched with 20 ml of HCl 1M; the separated organic layer was washed with a saturated solution of $NaHCO_3$, water until neutrality and finally brine. The organic phase was dried with anhydrous Na_2SO_4, filtered and the solvent evaporated to afford 520 mg of pale yellow solid **5**, which was used without any further purification (Yield = 86%). ^{1}H-NMR (300 MHz, $CDCl_3$) δ (ppm) = 8.59 (s, 1H), 8.45 (d, ^3J = 8.6 Hz, 1H), 8.11 (d, ^3J = 8.8 Hz, 1H), 8.07 (d, ^3J = 7.8 Hz, ^4J = 1.3 Hz, 1H), 7.90 (d, ^3J = 8.8 Hz, 1H), 7.87 (t, ^3J = 6.7 Hz, ^4J = 1.7 Hz, 1H), 7.80 (t, ^3J = 7.4 Hz, 1H), 5.57 (s, 2H), 4.55 (q, ^3J = 7.1 Hz, 2H), 1.51 (t, ^3J = 7.1 Hz, 3H). ^{13}C-NMR (75 MHz, $CDCl_3$) δ (ppm) = 164.6, 157.6, 138.5, 134.3, 133.4, 129.7, 129.0, 128.5, 128.2, 127.2, 126.2, 125.9, 123.9, 65.6, 62.0, 14.4.

This image cannot currently be displayed.

Figure S9. Compound 5 ^{1}H-NMR.

This image cannot currently be displayed.

Figure S10. Compound 5 ^{13}C-NMR.

Synthesis and characterization of compound 6. In a one neck round bottom balloon, 100 mg of **5** (0.36 mmol, 1 eq) were dissolved in 50 ml of chloroform. Then, 200 µl of triethylamine (1.08 mmol, 3 eq) and 45 µl of mesyl chloride (0.54 mmol, 1.5 eq) were respectively added. The reaction was kept at room temperature for 30 min, then 20 ml of a saturated solution of $NaHCO_3$ was added and the organic phase was extracted with water until neutrality. The solution was dried with anhydrous Na_2SO_4, filtered and the solvent evaporated to afford 140 mg of compound **6** as yellow solid, which was used without any further purification (Yield = quantitative). ^{1}H-NMR (300 MHz, $CDCl_3$) δ (ppm) = 8.62 (d, ^3J = 8.3 Hz, 1H), 8.60 (s, 1H), 8.06 (d, ^3J = 8.6 Hz, 1H), 8.04 (d, ^3J = 7.4 Hz, ^4J = 1.6 Hz, 1H), 7.86 (t, ^3J = 7.1 Hz, ^4J = 1.6 Hz, 1H), 7.83 (d, ^3J = 8.6 Hz, 1H), 7.79 (t, ^3J = 7.3Hz, ^4J = 1 Hz, 1H), 6.15 (s, 2H), 4.53 (q, ^3J = 7.2 Hz, 2H), 3.74 (s, 3H), 1.49 (t, ^3J = 7.2 Hz, 3H). ^{13}C-NMR (75 MHz, $CDCl_3$) δ (ppm) = 165.0, 151.4, 141.8, 138.4, 134.3, 133.1, 129.5, 128.6, 128.4, 128.1, 127.4, 127.2, 125.8, 124.5, 73.4, 61.9, 39.2, 14.4.

Figure S11. Compound 6 ^{1}H-NMR.

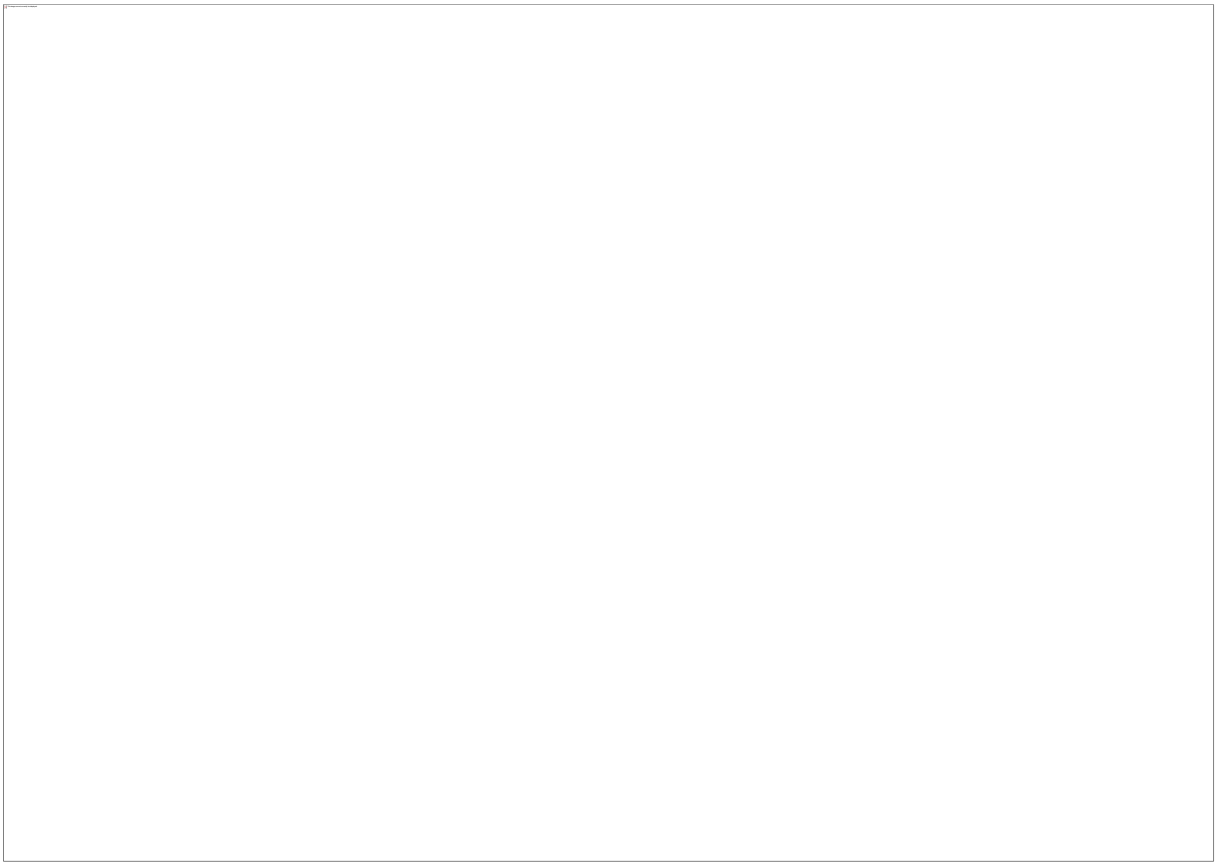

Figure S12. Compound 6 ^{13}C-NMR.

Synthesis and characterization of compound 7-Tris. In a two necks round bottom balloon, 56 mg of cyclen tetrahydrochloride (0.18 mmol, 1eq) were dissolved under nitrogen atmosphere in 100 ml of dry acetonitrile. Then, were respectively added 250 µl of N,N-diisopropylethylamine (1.41 mmol, 8 eq) and 250 mg of mesylate **6** (0.72 mmol, 4 eq). The reaction was refluxed under nitrogen atmosphere for 3 days. Then the solution was cooled down and the solvent evaporated. The crude product was purified by liquid chromatography (RP-C18 26g column, water with 0.1% TFA - acetonitrile gradient elution, on column sampling) to afford 70 mg of compound **7-Tris** as a brown glassy solid (Yield = 41%). ^{1}H-NMR (300 MHz, $CDCl_3$) δ (ppm) = 8.43-7.63 (m, 21H), 5.72-3.33 (m, 29H), 1.43-1.12 (m, 9H). ^{13}C-NMR (75 MHz, $CDCl_3$) δ (ppm) = 165.1, 164.6, 161.2, 160.7, 43.4, 140.2, 138.8, 138.0, 134.5, 134.4, 132.6, 129.8, 129.7, 129.3, 128.6, 127.8, 127.6, 127.3, 127.1, 126.9, 126.3, 125.2, 123.1, 118.0, 114.1, 61.5, 53.3, 49.1, 43.1, 29.7, 14.2, 13.9. Purity 98%, assessed by HPLC-MS (Jupiter Proteo analytical column), ret. time 10.3 min, mass spectrum (m/z), 963 $[M+H]^{+}$, 482 $[M+2H]^{2+}$.

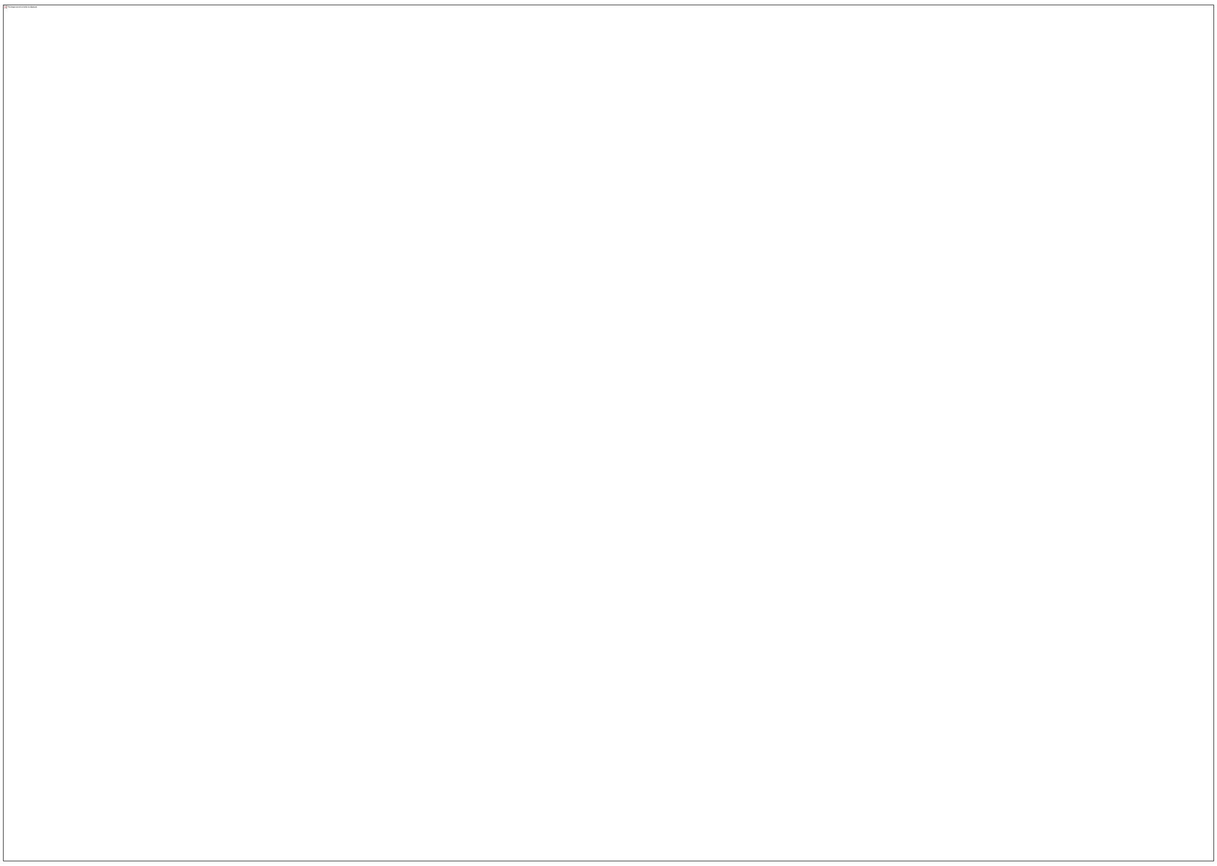

Figure S13. Compound 7-Tris ^{1}H-NMR spectrum.

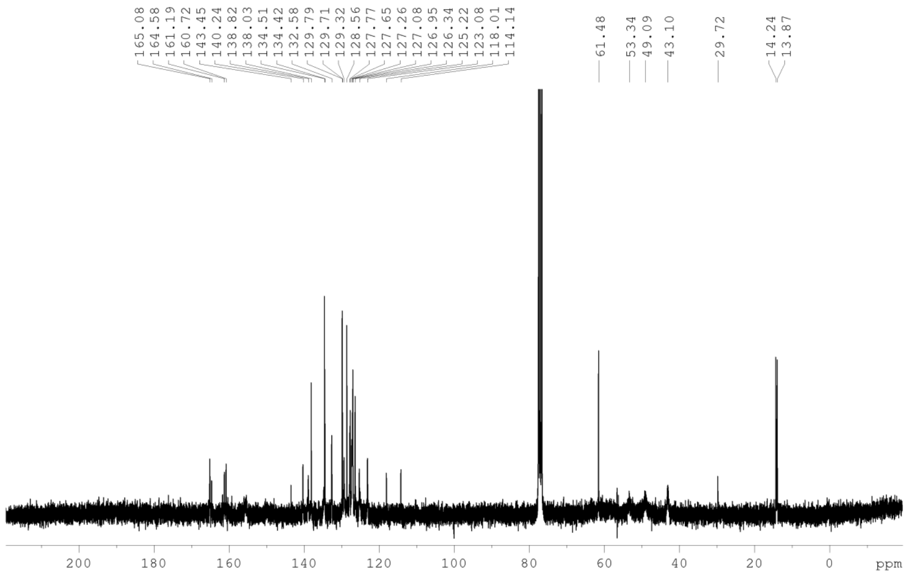

Figure S14. Compound 7-Tris ^{13}C-NMR spectrum.

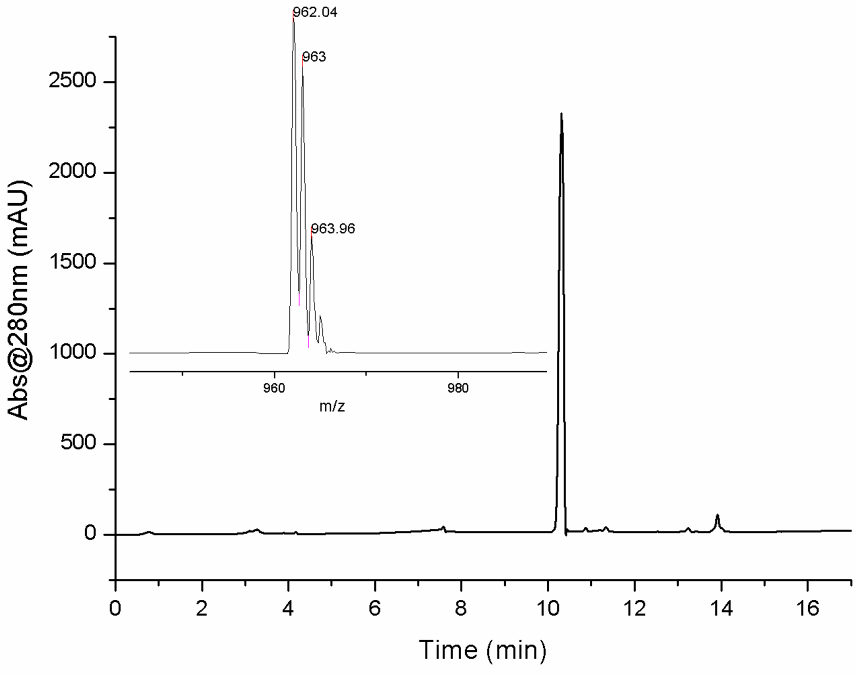

Figure S15. Compound 7-Tris HPLC and Mass spectra.

Synthesis and characterization of compound 7-Bis. In a two necks round bottom balloon, 56 mg of cyclen tetrahydrochloride (0.18 mmol, 1eq) were dissolved under nitrogen atmosphere in 30 ml of dry acetonitrile. Then, were respectively added 250 µl of N,N-diisopropylethylamine (1.41 mmol, 8 eq) and 190 mg of mesylate **6** (0.54 mmol, 3 eq). The reaction was refluxed under nitrogen atmosphere for 12 h. Then the solution was cooled down and the solvent evaporated. The crude product was purified by liquid chromatography (RP-C18 26g column, water with 0.1% TFA - acetonitrile gradient elution, on column sampling) to afford 40 mg of compound **7-Bis** (supposedly mix of two possible isomers) as a brown glassy solid (Yield = 45%). ^{1}H-NMR (300 MHz, $CDCl_3$) δ (ppm) = 7.94-7.84 (m, 6H), 7.71-7.54 (m, 6H), 7.21 (d, ^3J = 7.2 Hz, 2H), 4.47-3.06 (m, 24H), 1.47 (t, ^3J = 7.1 Hz, 6H). ^{13}C-NMR (75 MHz, $CDCl_3$) δ (ppm) = 165.6, 154.8, 139.4, 137.8, 134.4, 133.3, 129.6, 128.5, 128.2, 127.7, 127.4, 126.3, 125.3, 123.3, 62.7, 61.2, 55.4, 52.28, 44.0, 43.3, 14.1. Purity 97%, assessed by HPLC-MS (Jupiter Proteo analytical column), ret. time 9.5 min, mass spectrum (m/z), 699.1 $[M+H]^+$, 350 $[M+2H]^{2+}$.

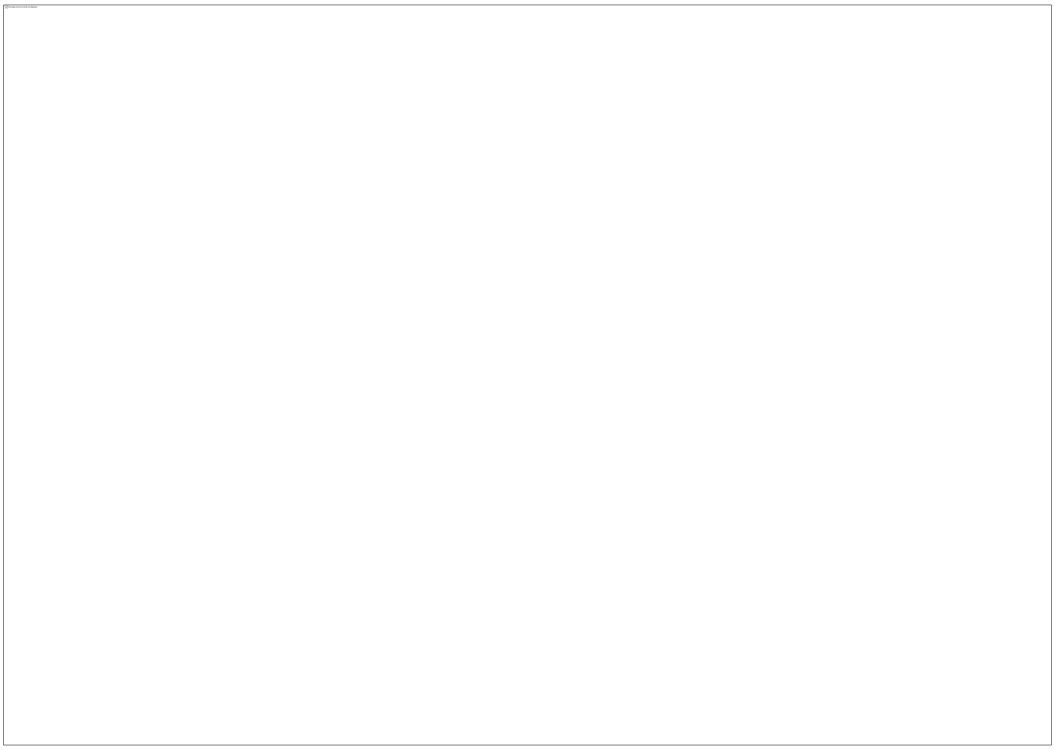

Figure S16. Compound 7-Bis ^{1}H-NMR spectrum.

Figure S17. Compound 7-Bis ^{13}C-NMR spectrum.

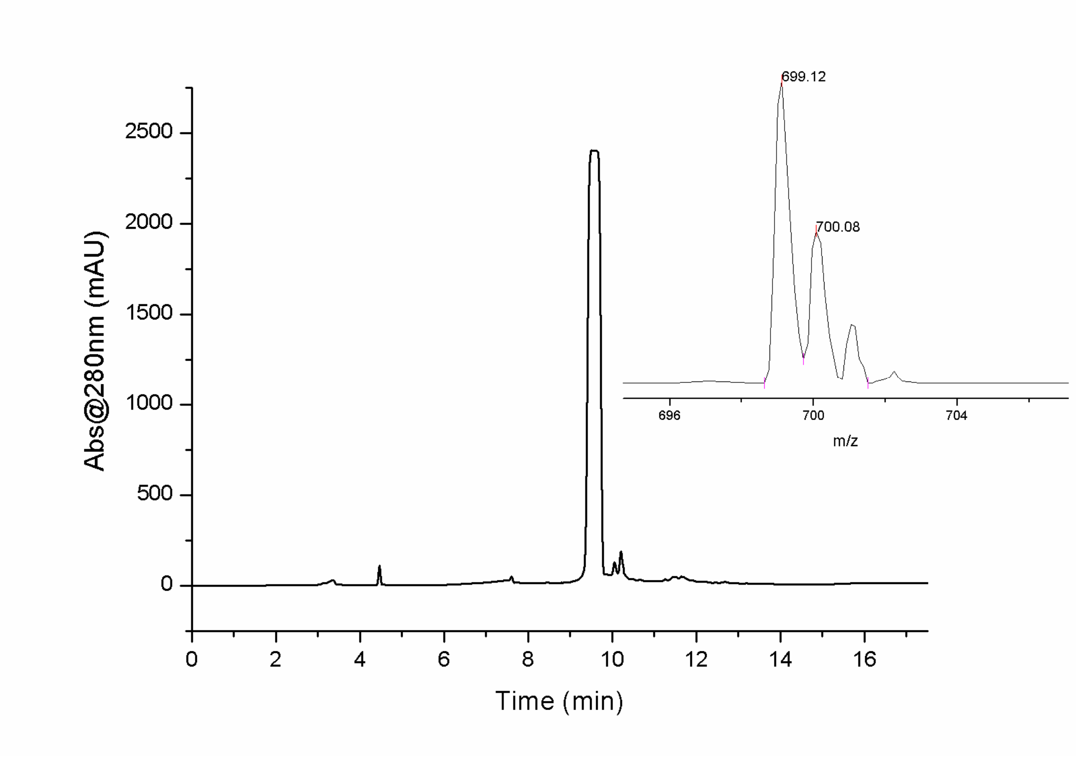

Figure S18. Compound 7-Bis HPLC and Mass spectra.

Synthesis and characterization of Tris-B[h]IQ. In a two necks round bottom balloon, 50 mg of compound **7-Tris** (0.05 mmol, 1 eq) were dissolved in 30 ml of dry acetonitrile under nitrogen atmosphere. Then, were respectively added 600 µl of N,N-diisopropylethylamine (3 mmol, 60 eq), 600 µl of ethyl 7-bromoheptanoate (3 mmol, 60 eq) and 450 mg of sodium iodide (3 mmol, 60 eq). The reaction was refluxed under nitrogen atmosphere for 3 days. Then, the solution was cooled down and the solvent evaporated. The residual solid was suspended in 50 ml of dichloromethane and the organic phase was washed with water and brine, dried with anhydrous sodium sulfate, filtered and the solvent evaporated. The crude product was purified by preparative HPLC (Proteo Semiprep, water with 0.01% TFA – acetonitrile with 5% water and 0.01% TFA, solvent gradient from 40% acetonitrile-based solvent to 100% in 7 min of a 12 min total run) to afford 30 mg of compound **Tris-B[*h*]IQ** as a brown glassy solid (Yield = 52%, purity 97% assessed by HPLC). ^{1}H-NMR (300 MHz, $CDCl_3$) δ (ppm) = 8.49-7.71 (m, 21H), 5.51-3.40 (m, 30H), 2.30-1.11 (m, 21H). ^{13}C-NMR (75 MHz, $CDCl_3$) δ (ppm) = 173.5, 164.9, 155.0, 143.5, 140.5, 139.7, 138.0, 134.4, 134.4, 132.7, 129.6, 129.5, 128.8, 128.4, 128.2, 127.9, 127.6, 127.2, 126.0, 125.7, 124.7, 122.9, 118.3, 114.5, 62.4, 61.7, 60.2, 52.7, 50.1, 48.2, 40.9, 33.8, 28.3, 26.2, 24.3, 14.1, 14.0. Purity assessed by HPLC-MS (Jupiter Proteo analytical column), ret. time 10.7 min, mass spectrum (m/z), 1118 $[M+H]^{+}$, 559.5 $[M+2H]^{2+}$.

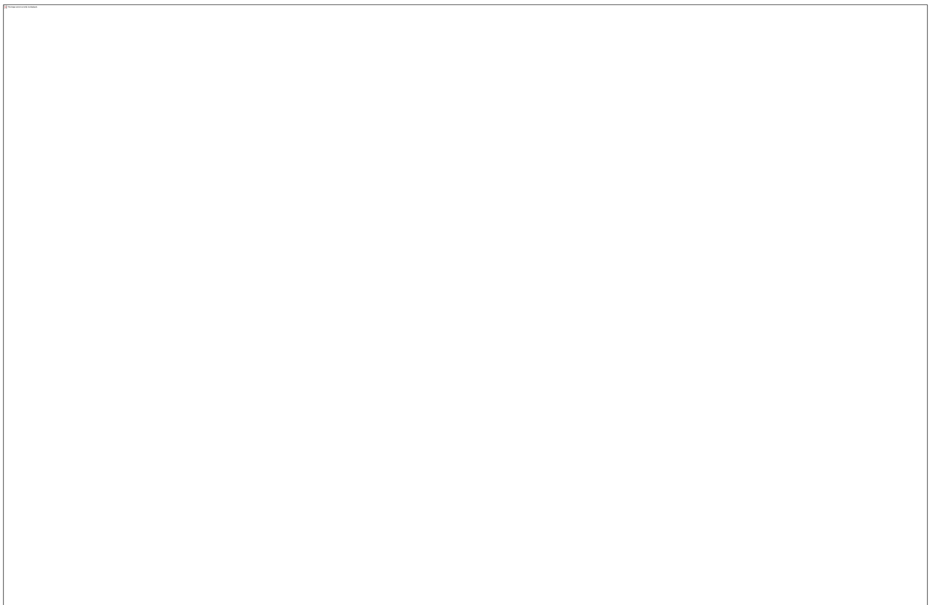

Figure S19. Compound Tris-B[*h*]IQ ^{1}H-NMR spectrum.

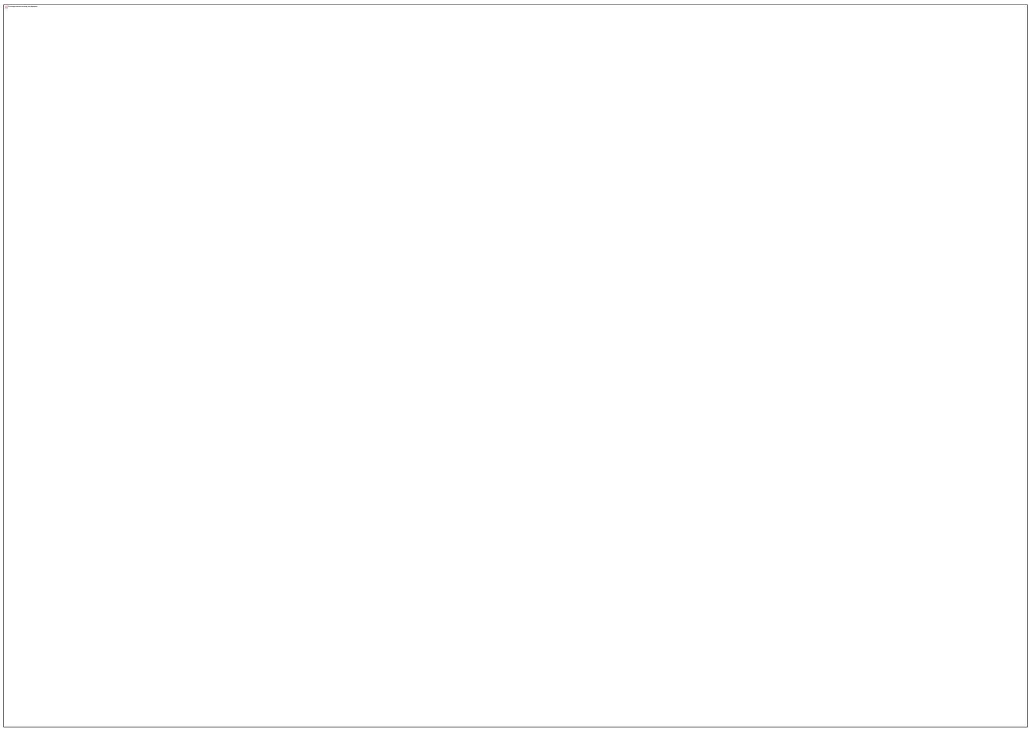

Figure S20. Compound Tris-B[*h*]IQ ^{13}C-NMR spectrum.

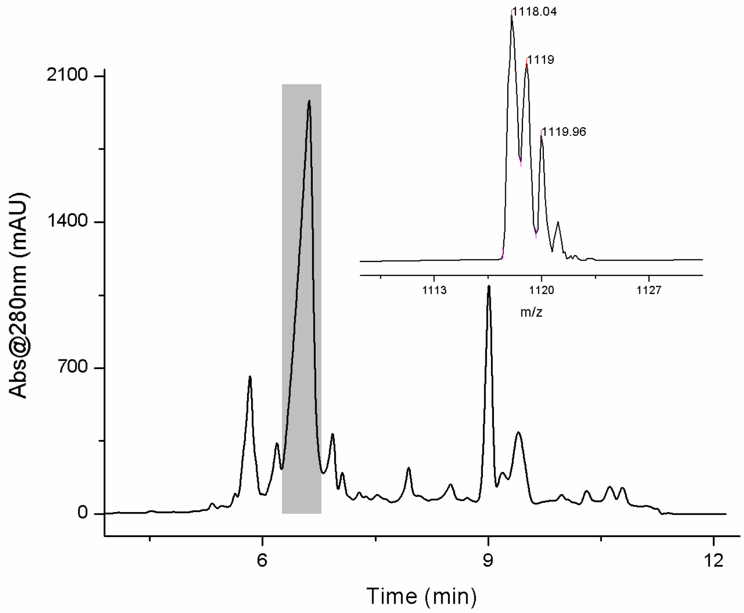

Figure S21. Preparative HPLC chromatogram with highlighted the fraction for product Tris-B[*h*]IQ together with the relative mass spectrum.

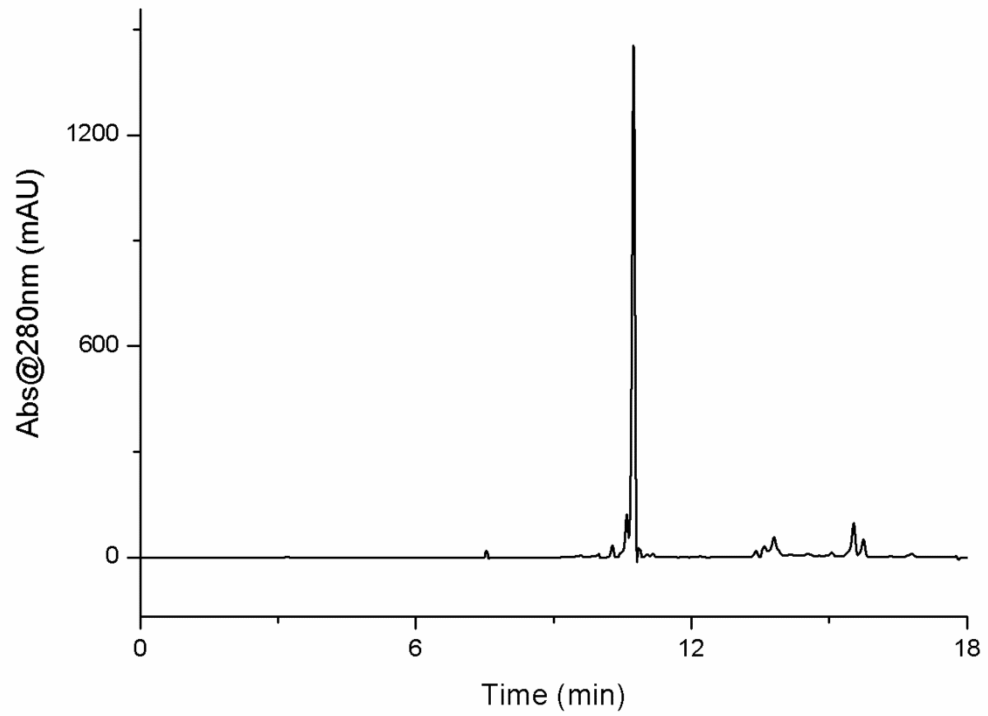

Figure S22. Compound Tris-B[*h*]IQ HPLC chromatogram after purification.

Synthesis and characterization of Na[Eu(Tris-B[h]IQ)]. In a one neck round bottom balloon, 10 mg of **Tris-B[*h*]IQ** (9µmol, 1 eq) were dissolved in 10 ml of a 1:1 mixture of water and ethanol. Then, 200µl of NaOH 1M (0.18 mmol, 20 eq) were added and the solution was refluxed until complete saponification (HPLC-MS followed). The ethanol was then evaporated under reduced pressure and the pH adjusted to 7 with HCl 1M. Finally 3.6 mg of $EuCl_3*6H_2O$ (9.9 µmol, 1.1 eq) were added and the final pH checked to be around 7. The solution was then concentrated to ca. 1 ml and the europium complex was purified (to get rid mainly of the salts) by Size Exclusion chromatography (LH-20, using water as eluent) to afford 4.6 mg of **Na[Eu(Tris-B[*h*]IQ)]** complex as pale yellow glassy solid (Yield = 46%). Purity 98%, assessed by HPLC-MS (Jupiter Proteo analytical column, 20 mM ammonium acetate/acetonitrile with a gradient 0 to 100% acetonitrile in 18 min): ret. time 7.2 min, mass spectrum (m/z) 1155 $[M+H]^+$, 577 $[M+2H]^{2+}$.

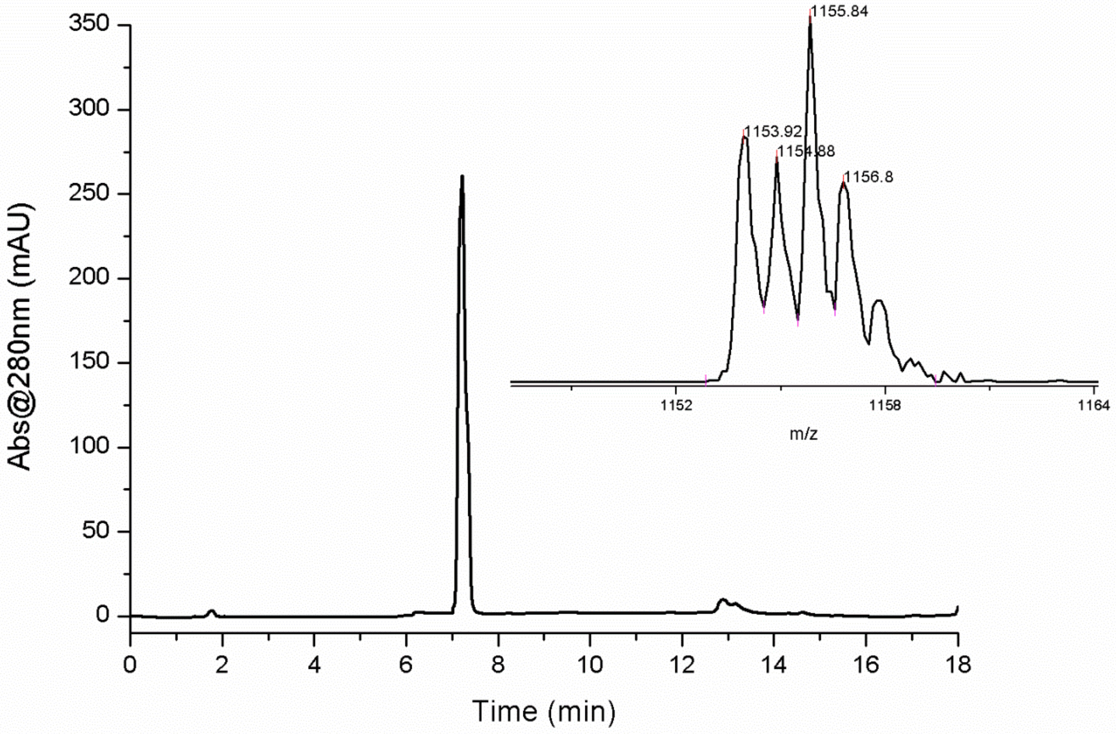

Figure S23. HPLC chromatogram of Na[Eu(Tris-B[h]IQ)] complex after purification with the relative mass spectrum.

Figure S24. Eu complex mass spectra comparison, experimental vs. calculated for $C_{60}H_{56}EuN_7O_8$.

Photophysical characterization of Na[Eu(Tris-B[h]IQ)]. Absorption and fluorescence spectra were recorded in cuvettes with 1 cm optical path (Hellma, Müllheim, Germany) at 23 ºC on a Jasco V550 spectrophotometer (Jasco, Easton, MD, USA) and a Cary Eclipse spectrofluorometer (Varian, Palo Alto, CA, USA), respectively. A stock solution of Na[Eu(Tris-B[h]IQ)] in DMSO (2 mM, as determined by ICP-MS), was diluted 1:200 (to 10 μM) in Tris-HCl buffer pH 7.4, and then absorbance and fluorescence were measured. The actual complex concentration was used to calculate the molar absorption spectrum of the complex (Figure S28).

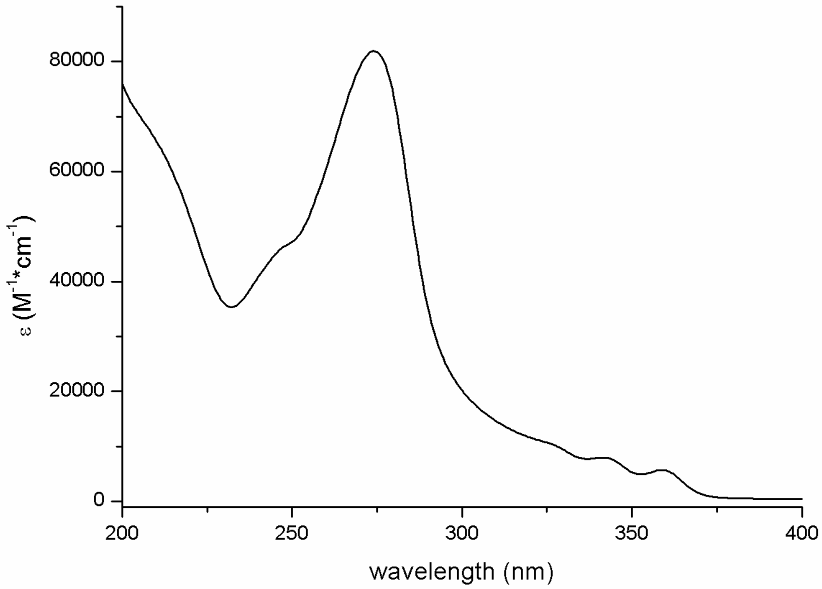

Figure S25. Molar absorption spectrum for Eu Complex.

The luminescence lifetime τ of the complex was measured by the phosphorescence plugin of the Cary-Eclipse spectrofluorometer. The luminescence decay was fitted to a monoexponential decay to recover τ (Figure S29).

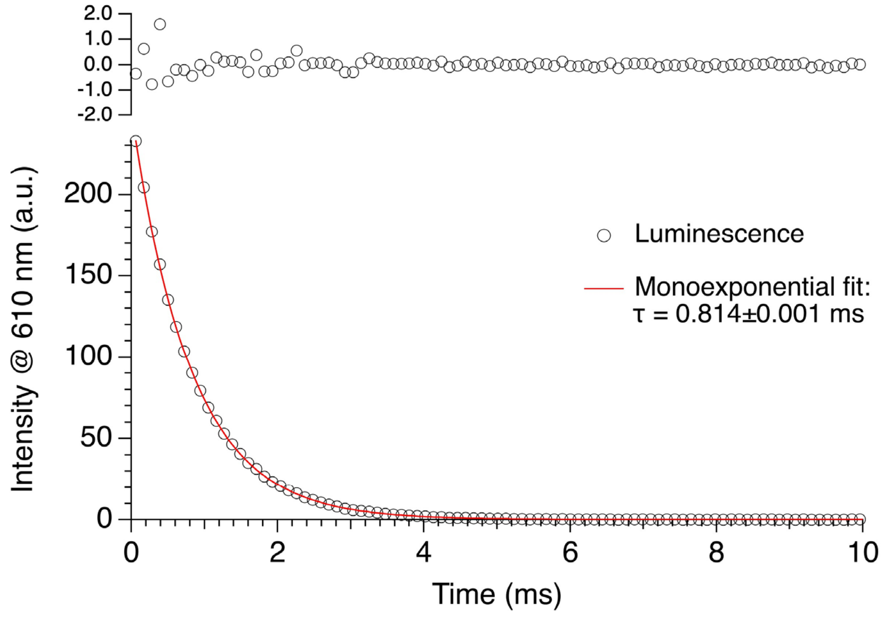

Figure S26. Luminescence lifetime decay for Eu complex.

For the determination of the quantum yield of the complex ($\Phi_{\mathrm{Eu}}^{\mathrm{L}}$), we measured the absorbance and fluorescence of several solutions of quinine sulfate in 0.5 M H_2SO_4 ($\Phi_{\mathrm{QS}} = 0.56$) and of the Eu complex in Tris-HCl pH 7.4 (Figure S30). Note that solution absorbances were in all cases below 0.1. Then, we linearly fitted the fluorescence *vs.* absorbance trends, to recover the two slopes m_{QS} and m_{Eu} (Figure S30). Finally, we calculated the complex quantum yield by the equation:

$$\Phi_{\mathrm{Eu}}^{\mathrm{L}} = \Phi_{QS} \cdot \frac{m_{\mathrm{Eu}}}{m_{\mathrm{QS}}}$$

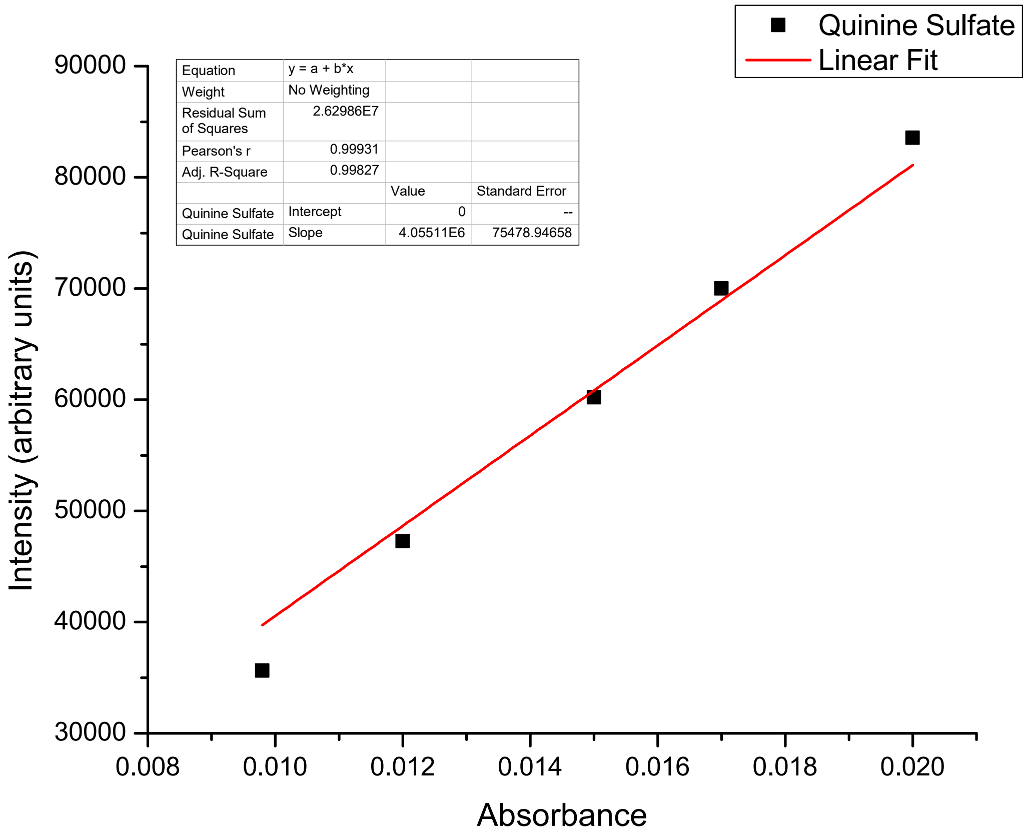

Quinine Sulfate
Linear Fit
Equation | y = a + b*x
Weight | No Weighting
Residual Sum of Squares | 2.62986E7
Pearson's r | 0.99931
Adj. R-Square | 0.99827
Value | Standard Error
Quinine Sulfate | Intercept | 0 | --
Quinine Sulfate | Slope | 4.05511E6 | 75478.94658
90000
80000
70000
60000
50000
40000
30000
Intensity (arbitrary units)
0.008
0.010
0.012
0.014
0.016
0.018
0.020
Absorbance

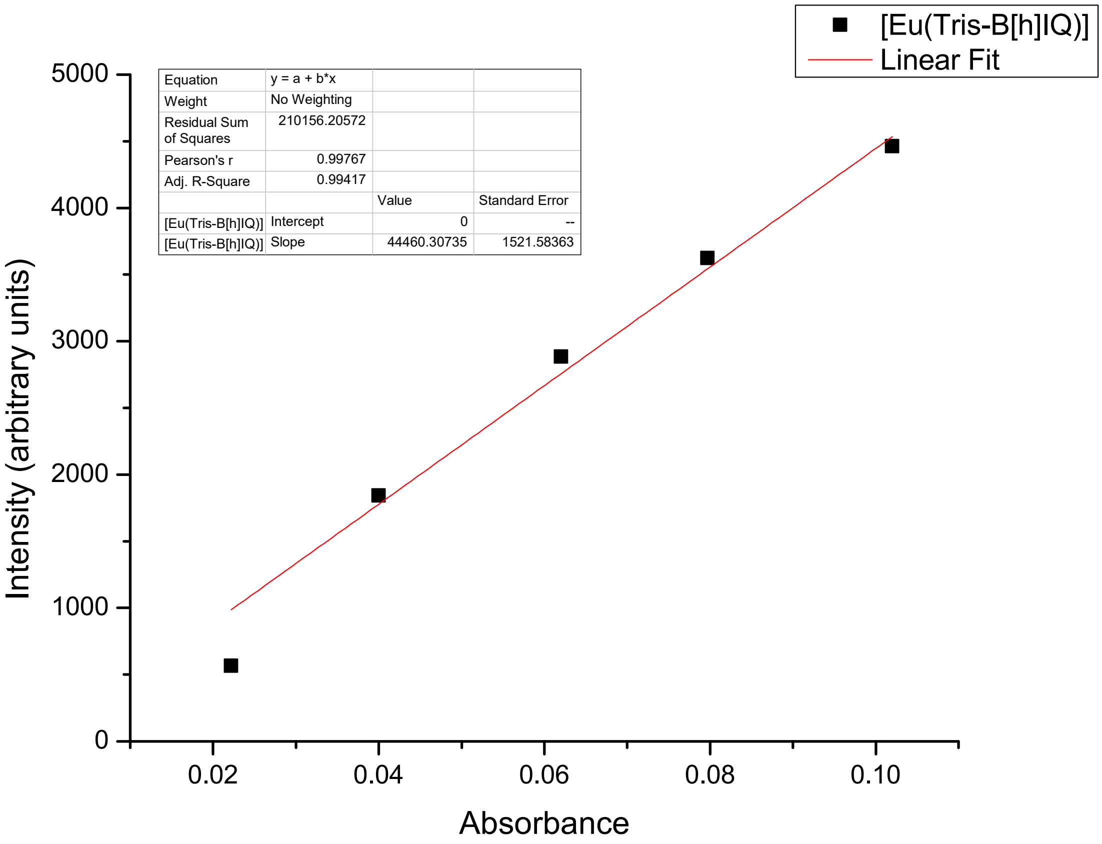

Figure S27. Determination of relative luminescence quantum yield.

Conjugation of Na[Eu(Tris-B[*h*]IQ)] with secondary IgG donkey anti rabbit antibody (Ab-Eu)

Complex **Na[Eu(Tris-B[*h*]IQ)]** was incubated at pH=6 with an excess (1000 fold mol/mol) of EDC and sulfo-NHS. Reaction was stirred overnight at 25 degrees and the obtained product was added (1000:1 molar ratio between the complex and the antibody) to an antibody solution (AffiniPure Donkey anti-Rabbit IgG H+L, Jackson ImmunoResearch) buffered at pH 7.1 and the resulting solution was stirred overnight at 4 °C, then dialyzed on centrifugal concentrators with a 100 kDa cutoff. Concentrated solution was repeatedly washed with PBS until no fluorophore was detectable in the filtrate. The conjugated antibody **Ab-Eu** was stored at 4 degrees until use, and

characterized by UV-VIS and fluorescence spectroscopy. Ratio between absorbance at 280 and 360 nm allowed determining a fluorophore/antibody ratio of 2.8.

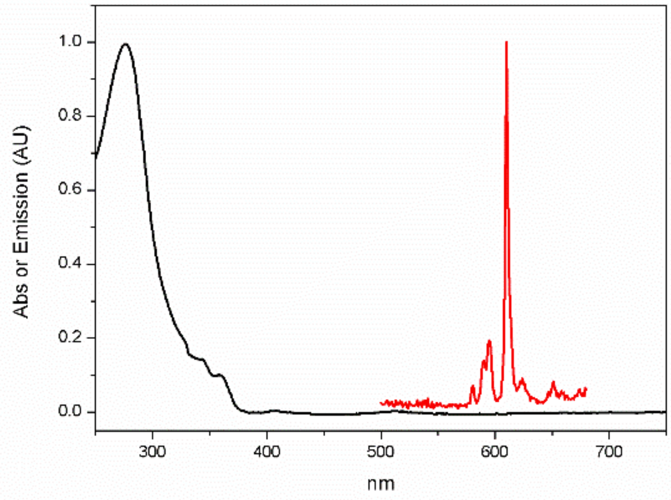

Figure S31: normalized absorption (black) and luminescence emission (red) of lanthanide labelled donkey anti-mouse IgG antibody.

Conjugation of Eu complex to the antibody was further confirmed by evaluating its retention time in GPC and the presence of Europium in the eluted fraction at the expected retention time for the antibody. A sample of labelled antibody (50 uL) was eluted and purified by size exclusion chromatography on a SuperdexTM 200 Increase 10/300 GL (V_0 = 8.05 mL) equilibrated with 10 mM phosphate buffer, pH 7.6, containing 250 mM sodium chloride and previously calibrated using a mixture of Ferritin (440 kDa), Aldolase (158 kDa) and Cytochrome C (12.4 kDa). The flow rate through the column was kept at 0.750 mL/min.. Retention time of the only peak absorbing at 280 nm is fully coherent with the expected molecular weight of the antibody (Figure S32). The eluted fraction containing the peak of interest was collected and digested in HCl 6M at 95°C for 12h. After digestion the sample was dried, reconstituted in HNO_3 2% in water for trace analysis and evaluated for Eu content by ICP-MS, comparing the result with a standard calibration curve. Results confirmed the presence of europium in the fraction containing the antibody ([Eu]=3.5 nM in the analysed sample).

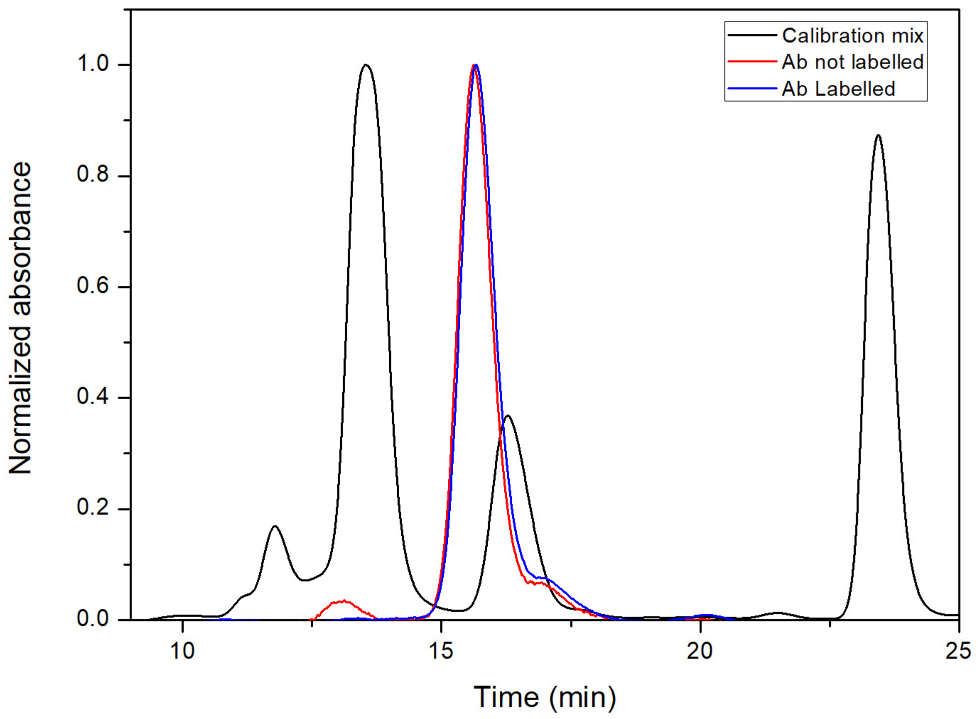

Figure S32: SEC chromatogram (λ 280nm) of a calibration protein mixture (black) and labelled (blue) or not labelled (red) antibody donkey anti-mouse IgG antibody. Maximum absorbance values are normalized to allow better comparison

Cell cultures

Adenocarcinoma human alveolar basal epithelial cells (A549) were grown in Roswell Park Memorial Institute (RPMI) 1640 medium (RPMI 1640, Invitrogen, Carlsbad, CA) supplemented with 10% Fetal Bovine Serum (FBS), glutamine (2mM), 100 U/ml penicillin and 100 mg/ml streptomycin (Invitrogen). Cells were maintained at 37°C in a humidified 5% CO_2 atmosphere. For live imaging, 6-7x10^4 cells were plated on a 35-mm glass bottom dish (Willco-dish HBST-3512/1.5-0.005) 24-48 hours before performing the immunofluorescence experiments.

Immunostaining

A549 cells were washed with phosphate buffer saline 1x (PBS, 3 times) and then fixed with paraformaldehyde (2% in PBS) for 15 min. After washing with PBS (3 times), cells were permeabilized with 0.1% Triton X-100 made in PBS, for 15 min. Cells were then washed with PBS (3 times), 0.5% Bovine Serum Albumin in PBS (PBB) 4 times), and exposed for 40 min to 2% Bovine Serum Albumin in PBS (BSA 2%). After washing with PBB (4 times), cells were incubated with Rabbit anti-human BMI1 monoclonal antibody (Cell Signaling Technologies #6964S; 1:600 dilution in PBB) for 1h at room temperature (RT) and additional 1.5 hours at 4 °C. Cells were washed with PBB (4 times), and incubated with the secondary antibody **Ab-Eu** at 1:100 dilution in PBB) for 1h at RT in dark. Next, cells were washed with PBB (4 times) and PBS (3 times). Cells were then maintained in PBS at 4 °C before imaging.

Fluorescence microscopy imaging

Fluorescence imaging was carried out by a Nikon TI2-E (Nikon Italia, Florence, Italy) inverted microscope interfaced with a ViCo-2 videoconfocal (Biomedica Mangoni, Pisa, Italy) structured illumination module. More specifically, the ViCo slider module was inserted into the field diaphragm of the microscope, so that the rectangular-shaped gratings could project on the sample a modulated excitation pattern. The microscope was also interfaced with an HG motorized Intensilight (Nikon Italia, Florence, Italy) as illumination source and a DS-Qi2 Mono Digital Microscope. **Ab-Eu** luminescence was excited by using a 360/20 filter (Semrock, USA). A TRITC-B-000 dichroic mirror (Semrock, USA) was used to separate out excitation from emission light. No emission filter was placed in the microscope, since the dichroic mirror allowed light with $\lambda > 570$ nm to reach the camera. Imaging was carried out by using a 63x CFI Plan Apochromat Lambda oil objective (Nikon Italia, Florence, Italy). Camera integration time was set in the range 0.5-1 s. Images were collected by NIS Element Ar (Nikon Italia, Florence, Italy) and then elaborated by ImageJ 1.51 (NIH, Bethesda, USA).

Computational methods and additional results

Geometry optimization of the analyzed structures was carried out at the density functional theory (DFT) level, using B3LYP as exchange and correlation (xc) potential, and a 6-31G(d) basis set, unless otherwise specified. For computational convenience the bioconjugable tether was replaced by a methyl group (see Figure S33). For the lanthanide ions we used the large-core quasi-relativistic effective core potential (ECP) from Stuttgart [1][2] and the related [5s4p3d]-GTO valence basis set. For comparison we also performed calculations employing the LANL2DZ (Los Alamos effective core potential plus double zeta) pseudopotential and basis set combination, substituting Eu with La, for which the pseudopotential is available [3]. The substitution with La has negligible effects on the lowest excitations (see Figure S35). The basis set data were taken from the basis-set exchange website www.basissetexchange.org [4] and from www.tc.uni-koeln.de/PP/index.en.html.

Excitation-energy calculations by time-dependent density functional theory (TD-DFT) where performed using the CAM-B3LYP exchange and correlation (xc) potential [5] and the same pseudopotential and basis sets reported above, unless otherwise specified. Implicit solvent effects were included using the polarizable continuum model [6] in the C-PCM variant [7] and the UAHF solute cavity [8].

The extinction coefficient ε(ν) was calculated as a sum of gaussian function centered on the (singlet) excitation energy and with a variance σ = 0.1eV (806.573 cm^{-1}) and height determined by the oscillator strength f_i of the excitation at wavenumber ν_i (in cm^{-1}), according to

$$\varepsilon(\nu) = 2.315 \cdot 10^{8} \frac{{f_L}^{2}}{n} \sum_{i} \frac{f_i}{\sqrt{2\pi}\sigma} e^{-\frac{1}{2}\left(\frac{\nu-\nu_i}{\sigma}\right)^{2}}$$

obtained from the relation between f and the integral of the extinction coefficient

$$f = \frac{1}{2.315 \cdot 10^{8}} \int \left(\frac{n}{{f_L}^{2}}\right) \varepsilon(\nu) d\nu$$

where ν is in cm^{-1} , n is the refraction index (1.33 for water) and f_L is the Lorentz local field factor, for which the optical value given by $f_L = (n^2 + 2)/3$ of (1.26 for water).

benzo[*h*]isoquinoline (B[*h*]IQ)

TD-DFT predicts that the lowest-energy excitation in benzo[*h*]isoquinoline is a π-π* excitation dominated by the HOMO → LUMO transition, at 300-336 nm depending on the xc functional used. Again, depending on the functional used one to three low oscillator strength excitations are present, and then two rather strong (f ~ 0.7-1.1, where f is the oscillator strength of the excitation) excitations, located around 290-260 nm. CAM-B3LYP is the most blue shifted of the three, while B3LYP is closer to the experimental value for the lower energy absorption band at 360 nm. However, CAM-B3LYP is more reliable when dealing with charge-transfer excitations.

Table S1: Lowest energy excitation in the intermediate benzo[*h*]isoquinoline system

XC functional	PCM solvent	Basis set	nm	f	transitions	
CAM-B3LYP[§]	Gas-phase	6-31G*	295	0.04	HOMO → LUMO	0.54
					$HOMO^{-1}$ → $LUMO^{+1}$	0.40
					$HOMO^{-1}$ → LUMO	-0.10
CAM-B3LYP	DCM	6-31+G*	300	0.21	HOMO → LUMO	0.58
					$HOMO^{-1}$ → $LUMO^{+1}$	0.31
					$HOMO^{-1}$ → LUMO	0.20
PBE0	DCM	6-31+G*	324	0.14	HOMO → LUMO	0.63
					$HOMO^{-1}$ → $LUMO^{+1}$	-0.28
					$HOMO^{-1}$ → LUMO	0.12
B3LYP	DCM	6-31+G*	336	0.12	HOMO → LUMO	0.64
					$HOMO^{-1}$ → $LUMO^{+1}$	0.26

§ B3LYP/6-31G* geometry

All others are on geometries optimized with the same xc potential/PCM/basis set combination as for the excitation energy calculation

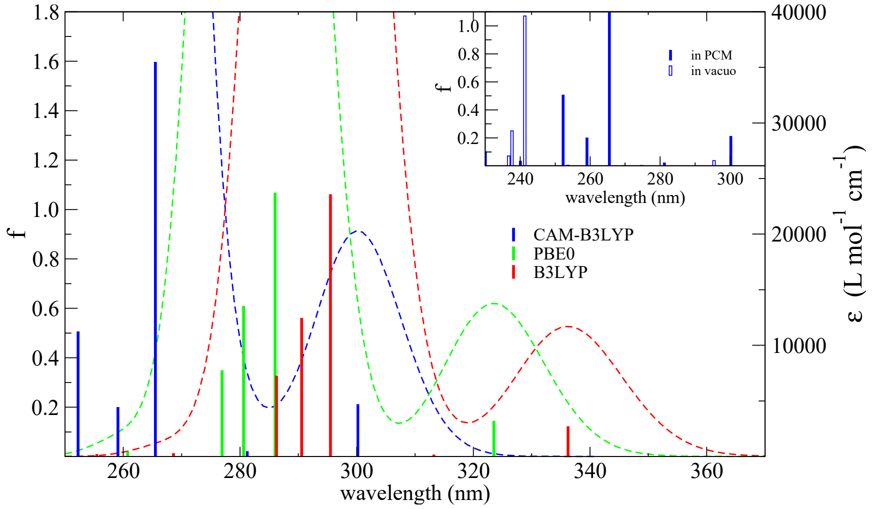

Figure S33: TD-DFT excitation energies of **B[*h*]IQ** for the various functional employed (PCM solvent is DCM and basis set is 6-31G(d)). The height of the bar corresponds to the oscillator strength (f) of each excitation. The dashed lines is a sum of gaussian functions centered at each excitation wavelength, with height proportional to f and broadened by a conventional value of 0.1 eV (~800 cm^{-1}). The inset shows the comparison between the PCM and in vacuo CAM-B3LYP results.

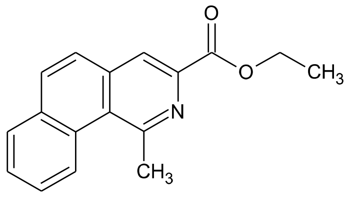

Figure S34: Molecular orbitals involved in the excitations.

Eu/La(Tris-B[*h*]IQ)

CAM-B3LYP in PCM fails to converge with the combination of basis-set and Eu/La Stuttgart pseudopotentials. However using the LANL2 pseudopotential available for La the calculations converged. Using LANL pseudopotential instead of Stuttgart's leads to a few-nm blue shift for the excitations of La(**Tris-B[*h*]IQ)** complex in the gas phase (Figure S34). The calculation including PCM (water) reveals an increase in the lower energy band intensity. Overall, implicit solvent effects do not significantly modify the low-lying excitation energies.

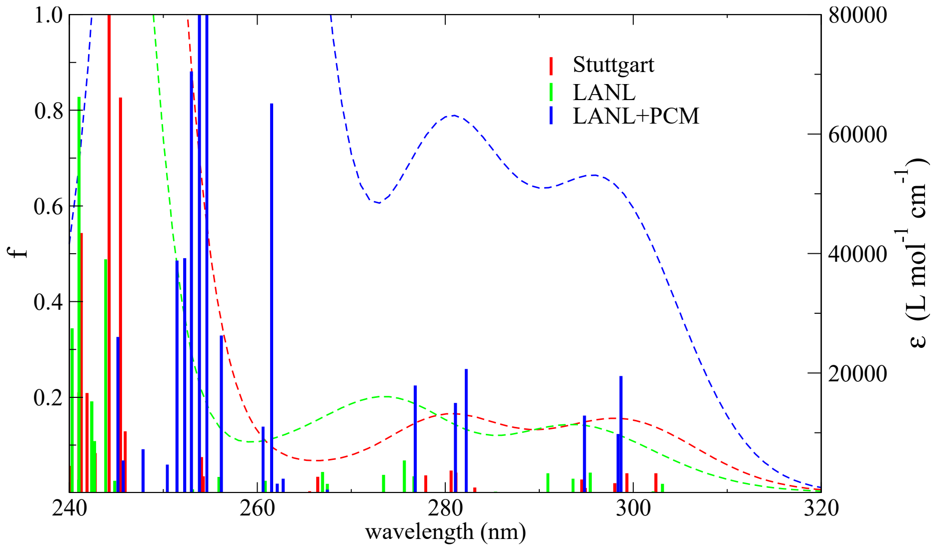

Figure S35: Effect of different choices of pseudopotential for La and of implicit solvent (water) on the excitations of the **La(Tris-B[*h*]IQ)** complex. Red: Stuttgart pseudopotential; green: LANL pseudopotential; blue: LANL pseudopotential in PCM.

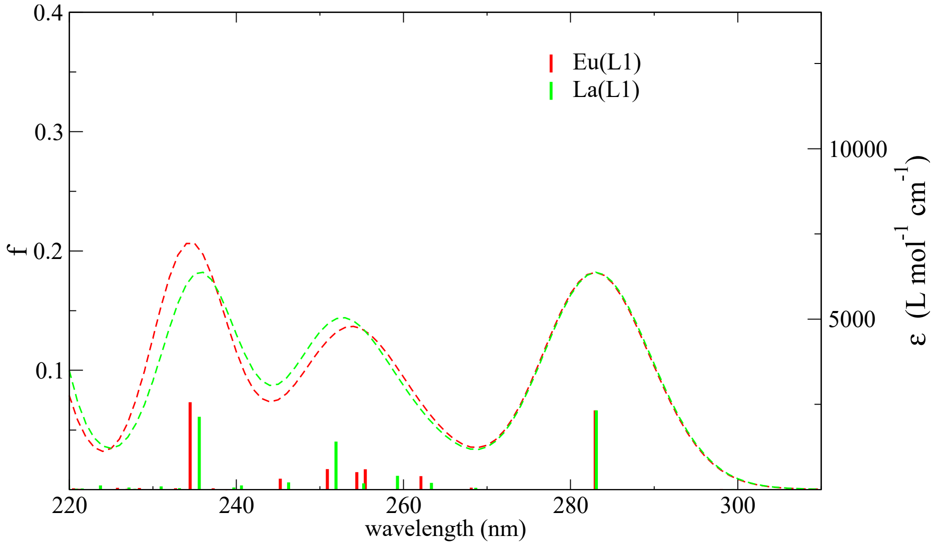

Figure S36: Effect of replacing Eu with La on the excitations of the Ln(L1) complex. The same Ln(L1) structure was employed in the two TD-DFT calculations.

References

1. Dolg M, Stoll H, Savin A, Preuss H (1989) Energy-adjusted pseudopotentials for the rare earth elements. Theor Chim Acta 75: 173–194. doi:10.1007/BF00528565.

2. Dolg M, Stoll H, Preuss H (1993) A combination of quasirelativistic pseudopotential and ligand field calculations for lanthanoid compounds. Theor Chim Acta 85: 441–450. doi:10.1007/BF01112983.

3. Hay PJ, Wadt WR (1985) Ab initio effective core potentials for molecular calculations. Potentials for K to Au including the outermost core orbitals. J Chem Phys 82: 299–310. Available: http://aip.scitation.org/doi/10.1063/1.448975.

4. Schuchardt KL, Didier BT, Elsethagen T, Sun L, Gurumoorthi V, et al. (2007) Basis Set Exchange: A Community Database for Computational Sciences. J Chem Inf Model 47: 1045–1052. Available: https://pubs.acs.org/doi/10.1021/ci600510j.

5. Yanai T, Tew DP, Handy NC (2004) A new hybrid exchange-correlation functional using the Coulomb-attenuating method (CAM-B3LYP). Chem Phys Lett 393: 51–57. Available: http://www.sciencedirect.com/science/article/pii/S0009261404008620.

6. Tomasi J, Mennucci B, Cammi R (2005) Quantum Mechanical Continuum Solvation Models. Chem Rev 105: 2999–3094. Available: https://pubs.acs.org/doi/10.1021/cr9904009.

7. Cossi M, Rega N, Scalmani G, Barone V (2003) Energies, structures, and electronic properties of molecules in solution with the C-PCM solvation model. J Comput Chem 24: 669–681. Available: http://doi.wiley.com/10.1002/jcc.10189.

8. Barone V, Cossi M, Tomasi J (1997) A new definition of cavities for the computation of solvation free energies by the polarizable continuum model. J Chem Phys 107: 3210–3221. Available: http://aip.scitation.org/doi/10.1063/1.474671.
